# Multi-Omic Bicluster Association Analysis (MOBAA)—a tool for identifying population subgroups with distinct multi-omics molecular profiles

**DOI:** 10.1093/bioadv/vbag156

**Published:** 2026-06-03

**Authors:** Binisha H Mishra, Pashupati P Mishra

**Affiliations:** Department of Clinical Chemistry, Faculty of Medicine and Health Technology, Tampere University, 33520 Tampere, Finland; Finnish Cardiovascular Research Center Tampere, Faculty of Medicine and Health Technology, Tampere University, 33520 Tampere, Finland; Department of Clinical Chemistry, Fimlab Laboratories, 33520 Tampere, Finland; Tampere University Hospital, Wellbeing Services County of Pirkanmaa, 33520 Tampere, Finland; Department of Clinical Chemistry, Faculty of Medicine and Health Technology, Tampere University, 33520 Tampere, Finland; Finnish Cardiovascular Research Center Tampere, Faculty of Medicine and Health Technology, Tampere University, 33520 Tampere, Finland; Department of Clinical Chemistry, Fimlab Laboratories, 33520 Tampere, Finland; Tampere University Hospital, Wellbeing Services County of Pirkanmaa, 33520 Tampere, Finland

## Abstract

**Motivation:**

The increasing availability of multi-omic datasets from the same individuals presents the opportunity to uncover distinct molecular profiles across subgroups within a study population. These profiles may be linked to specific biological traits, such as disease status, and have distinct disease trajectories. Importantly, they could reveal robust, multi-layered molecular signatures with potential applications in early diagnosis, prognosis, and treatment advancing precision medicine. Although several integrative multi-omic methods have been developed in recent years, most are tailored to population-level analyses and are not well-suited for identifying signals specific to subpopulations.

**Results:**

We developed MOBAA (Multi-Omic Bicluster Association Analysis), a novel data-driven integrative machine-learning framework for identifying subgroups within a study population that exhibit distinct multi-omic molecular profiles. MOBAA is scalable and capable of handling multiple omics simultaneously without relying on parametric distributional assumptions. It combines biclustering algorithms with hierarchical clustering-based module identification and uses permutation-derived empirical *P*-values. This approach provides a comprehensive and intuitive view of underlying biological variation and population heterogeneity facilitating discovery of complex, multi-layered molecular signatures.

**Availability and implementation:**

The code is available as MOBAA R package. All source code as well as comprehensive documentation and examples are provided at https://github.com/pmishra912/MOBAA.

## 1. Introduction

Identifying subgroups within a population that exhibit distinct biological characteristics is critical for advancing precision medicine. The emergence of omic technologies has enabled the generation of large-scale, multi-layered molecular datasets, offering unprecedented opportunities to uncover population subgroups defined by unique multi-omic profiles. Individuals within these subgroups may exhibit distinct disease trajectories and differential responses to the same treatments.

Multi-omic-based subgroup identification is particularly valuable for complex and heterogeneous diseases such as cancer and cardiometabolic disorders. These diseases often present with variable clinical manifestations, drug resistance, and treatment failure. Identifying disease subtypes based on multi-omic data can support the development of tailored therapeutic strategies and improve treatment outcomes. For instance, discovering a subgroup of cancer patients with a unique multi-omic signature predictive of a distinct treatment response can inform the design of targeted therapies for that subgroup.

Several integrative multi-omics methods, such as MOFA2 (multi-omic factor analysis v2) (Argelaguet *et al*. 2020) and iClusterPlus (Mo *et al*. 2018), can identify latent factors that capture shared variation across omics layers and can be used to infer sample subgroups. However, these approaches rely on probabilistic modeling and parametric distributional assumptions, and biomarker discovery is typically indirect, performed by examining feature loadings or coefficients associated with latent factors. To address these limitations, we propose MOBAA (Multi-Omic Bicluster Association Analysis), a modular biclustering-based integrative pipeline for identifying population subgroups with distinct molecular profiles. MOBAA first applies an ensemble biclustering strategy independently to each omic dataset, and then groups biclusters across modalities into “bicluster modules” based on sample overlap quantified using the Jaccard index. Modules are defined by a similarity threshold that ensures high sample concordance among their constituent biclusters. Within each module, the bicluster with the greatest sample overlap is retained, and its statistical significance is evaluated through permutation-based empirical *P*-values. While a related concept was introduced by (Arnedo *et al*. 2013), our approach extends it by incorporating ensemble biclustering and enabling scalable integration of more than two omic layers through Jaccard-based module construction. Consequently, MOBAA provides a fully data-driven, distribution-free framework that simultaneously identifies subgroups and the multi-omic features that characterize them, offering a complementary alternative to existing latent-factor-based integrative methods.

## 2. Methods

### 2.1 Biclustering omic data matrices

Biclustering, also known as co-clustering or two-way clustering, is an unsupervised learning technique that simultaneously partitions the rows and columns of a data matrix to identify submatrices called biclusters that exhibit locally coherent patterns. In the context of omics data, such as gene expression matrices, biclustering can reveal subsets of features (e.g. genes) that display coordinated behavior across specific subsets of samples or conditions. This enables the identification of biologically meaningful subpopulations, such as disease subtypes or condition-specific molecular modules, which may be overlooked by traditional one-dimensional clustering methods.

**Figure 1:**
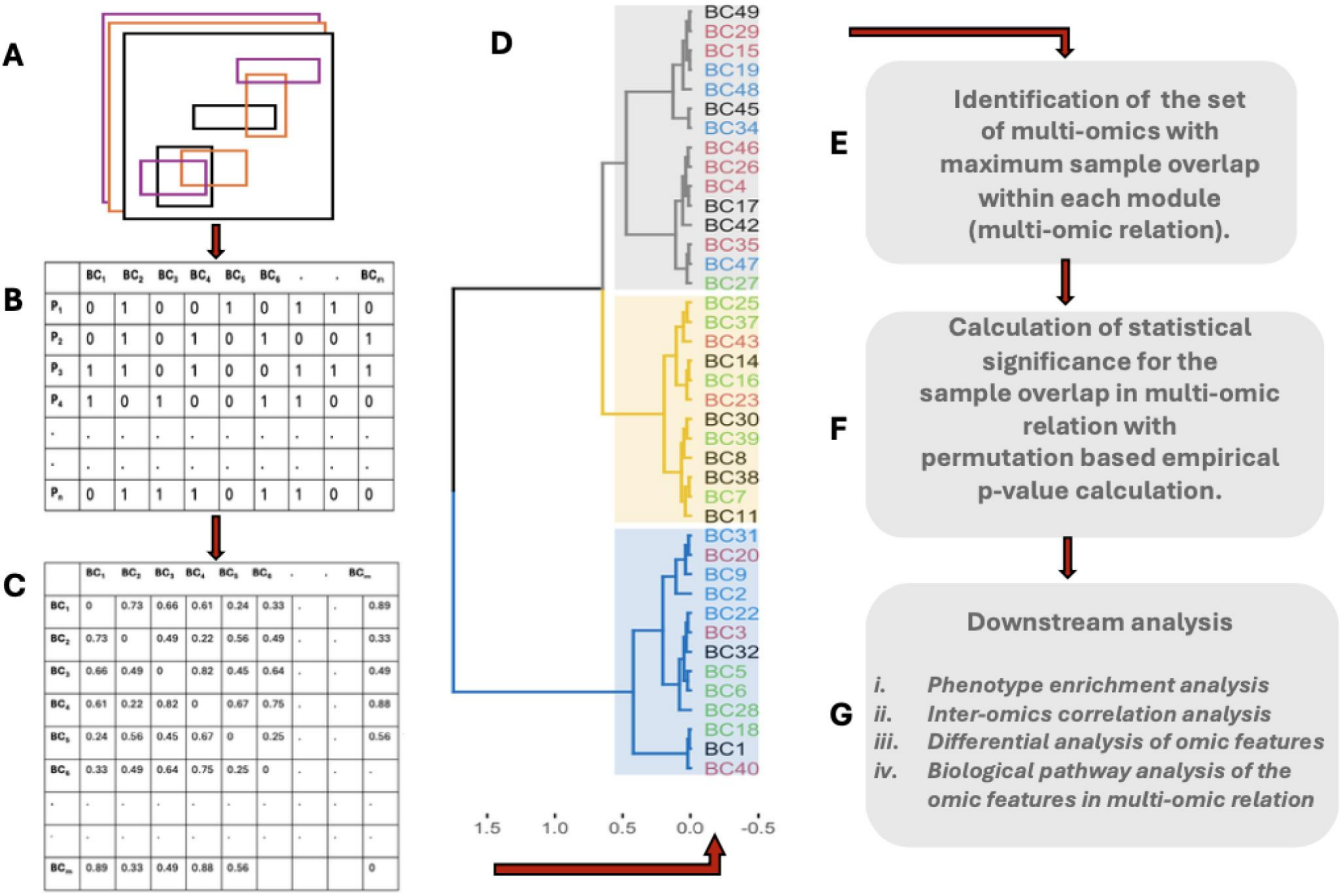
Schematic diagram representing the steps involved in MOBAA. **A**. Multialgorithm ensemble biclustering of individual omics separately. **B**. Compilation of all resulting biclusters into a unified binary presence–absence matrix with rows representing study participants and columns representing individual biclusters. **C**. Bicluster distance matrix derived from pairwise Jaccard similarity between the biclusters. **D**. Hierarchical clustering of the biclusters to identify groups of biclusters sharing large number of study participants (bicluster modules). Biclusters from different omics are represented by different colors. **E**. Identification of set of biclusters, one from each omic, such that the intersection of their associated participants is maximized (multi-omic relation). **F**. Calculation of statistical significance for the sample overlap in the multi-omic relation with permutation-based empirical *P*-value calculation. **G**. Downstream analysis of multi-omic relation including, phenotype enrichment analysis, inter-omics correlation analysis of the omic features, differential analysis of omic features in the multi-omic relation between the samples in the relation and in the rest of the omic data, biological pathway analysis.

Our proposed pipeline, MOBAA involves biclustering of individual omics data matrices independently, followed by integrative analysis to identify coherent multi-omic subgroups (Fig. 1). Biclustering is conducted using a multialgorithm ensemble approach involving multiple established algorithms such as FABIA (Hochreiter *et al*. 2010), QUBIC (Zhang *et al*. 2017), Plaid (Lazzeroni *et al*. 2002), and ISA (Bergmann *et al*. 2003), as implemented in the *mosbi* R/Bioconductor package (Rose *et al*. 2022). MOSBi (molecular signature identification using biclustering) is an ensemble biclustering framework that integrates results from multiple biclustering algorithms. It constructs a network of bicluster overlaps with nodes representing individual biclusters and edges representing sample overlap between them. To reduce noise and remove spurious bicluster similarity, an error model is applied to filter edges. Louvain community detection is then used to identify robust consensus biclusters, capturing consistent sample and feature modules across heterogenous omics datasets (Rose *et al*. 2022).

### 2.2 Bicluster module identification

Following biclustering on each omics dataset, we compile all resulting biclusters into a unified binary presence–absence matrix, where rows represent study participants and columns correspond to individual biclusters. A value of 1 indicates that a participant belongs to a bicluster; a 0 indicates absence. To assess the similarity between biclusters within as well as across omics layers, we compute the pairwise Jaccard similarity between the columns of this matrix, quantifying the degree of sample overlap:


$$J\left(A,\;B\right)=\frac{\left|A\cap B\right|}{\left|A\cup B\right|}$$

where |A ∩ B| is the number of study participants shared between biclusters A and B, and |A ∪ B| is the total number of unique study participants in biclusters A and B. MOBAA assumes that all omics layers are matched across a common set of samples. In cases where sample availability is imbalanced across modalities, users are advised to pre-filter or impute missing data to ensure that the unified presence–absence matrix reflects a consistent sample universe for reliable Jaccard-based module identification. The distance matrix derived from the resulting similarity matrix is used to group biclusters with overlapping sample composition into coherent bicluster modules. This grouping is performed using hierarchical clustering and dynamic tree cut algorithm as implemented in the WGCNA R package (Langfelder *et al*. 2008) based on user-provided threshold for minimum module size (number of biclusters) and distance. The choice of these parameters in hierarchical clustering of biclusters influence module granularity and robustness; higher cut heights and larger minimum module sizes yield more conservative, larger modules, while lower cut heights and smaller sizes allow finer subdivision at the cost of potential noise. Each bicluster module represents a set of biclusters that share a similar subset of study participants and may reflect coordinated biological processes or multi-omic molecular signatures.

### 2.3 Multi-omic relations

Bicluster modules often contain multiple biclusters from the same omic layer as well as from others, necessitating further filtering to identify meaningful cross-omic relationships. Within each module, we identify a subset consisting of one bicluster per omic layer such that the overlap in study participants among them is maximized. We refer to this subset as a multi-omic relation, representing a set of biclusters, one from each omic, that are most strongly connected through shared sample membership. To assess the statistical significance of each multi-omic relation, we employ a permutation-based approach that estimates the probability of observing the degree of sample overlap by chance.

### 2.4 Downstream analysis of multi-omic relations

Statistically significant multi-omic relations identified by MOBAA represent biologically distinct population subgroups characterized by coherent multi-layer molecular profiles. A key next step is to determine whether these molecularly defined subgroups are associated with specific phenotypes or clinical outcomes. Such associations can help identify disease-specific or phenotype-specific biomarkers and improve stratification strategies in precision medicine.

To assess phenotype enrichment, MOBAA performs a proportion test (prop.test in R) to evaluate whether the subgroup defined by a multi-omic relation exhibits a statistically significant overrepresentation of a particular diagnosis or phenotype compared to the rest of the cohort.

MOBAA also enables pairwise correlation analysis of omic features across biclusters within a multi-omic relation, at both the summary level and the feature level. At the summary level, the first principal component (PC1) from a principal component analysis (PCA) is used as a representative profile for each bicluster. Correlations between these PC1 profiles across omics can highlight coordinated variation. At the feature level, correlations between individual features (e.g. genes, proteins, CpGs) across omics can reveal potential regulatory or mechanistic links.

To further characterize the molecular signatures of each bicluster within a relation, MOBAA conducts differential analysis (e.g. expression, methylation, or abundance) by comparing individuals within the bicluster to all others in the dataset. This identifies omic features that are significantly upregulated, downregulated, or otherwise altered in the subgroup. Statistical testing is performed using either Student’s t-test or the Mann–Whitney U test, depending on data distribution. Results for selected features can be visualized using boxplots, enabling intuitive interpretation of molecular differences.

For functional interpretation, biological pathway enrichment analysis can be performed on the differentially altered features using tools such as *clusterProfiler* (Xu *et al*. 2024) for gene ontology and pathway enrichment, and *missMethyl* (Phipson *et al*. 2015) for methylation-specific analyses.

### 2.5 Benchmarking MOBAA against state-of-the-art methods on real and simulated overlapping multi-omics data

**Figure 2:**
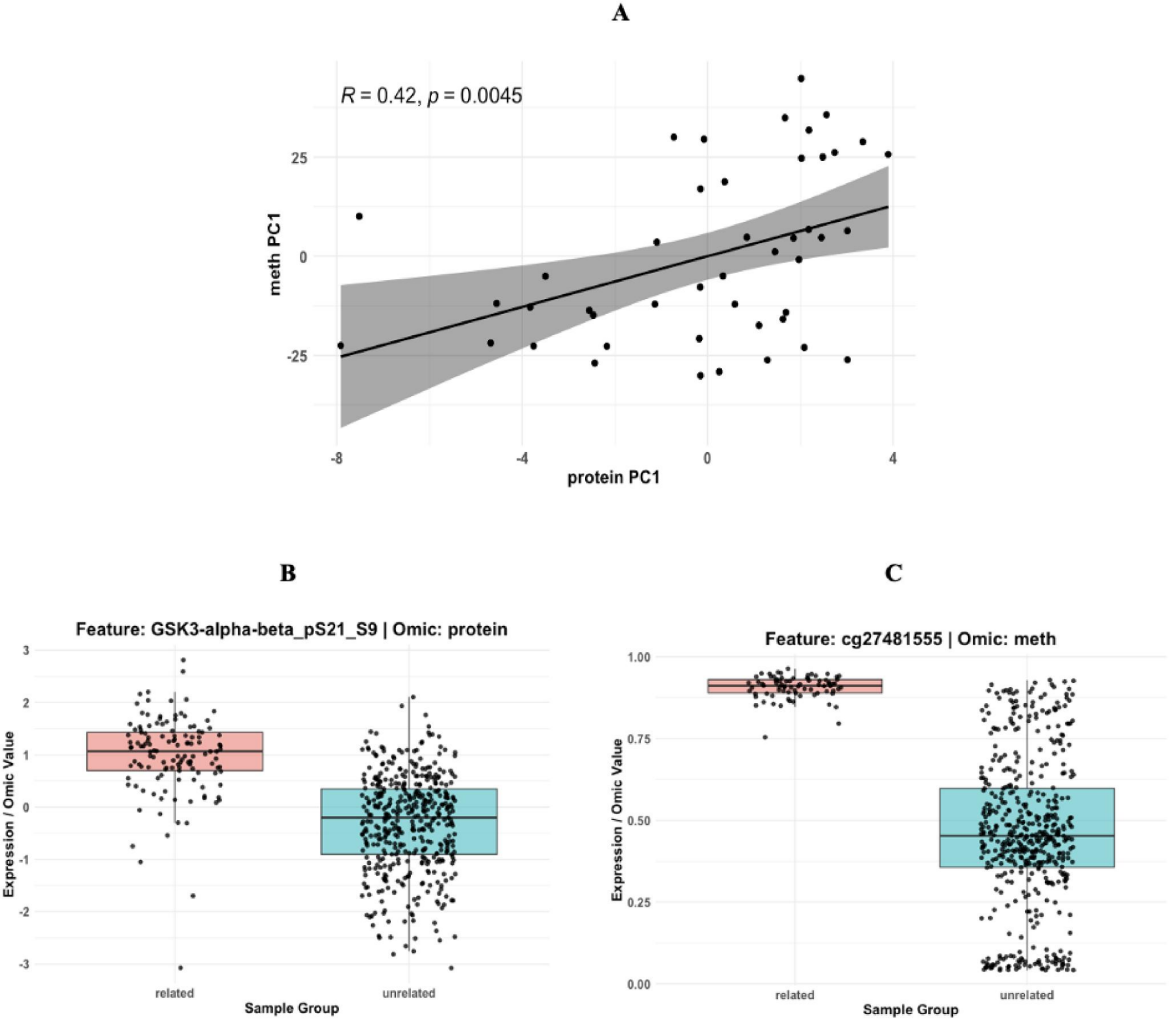
A. Correlation between eigenfeatures (first principal component) of DNA methylation and protein expression from the corresponding biclusters in the turquoise multi-omic relations. **B**. Boxplot showing differences in expression levels of the top-ranked protein and CpG site between participants within the turquoise multi-omic relation (“related”) and those outside the relation in the rest of the cohort (“unrelated”). **C**. Boxplot showing differences in methylation levels of the top-ranked CpG site between related and unrelated participants.

**Figure 3:**
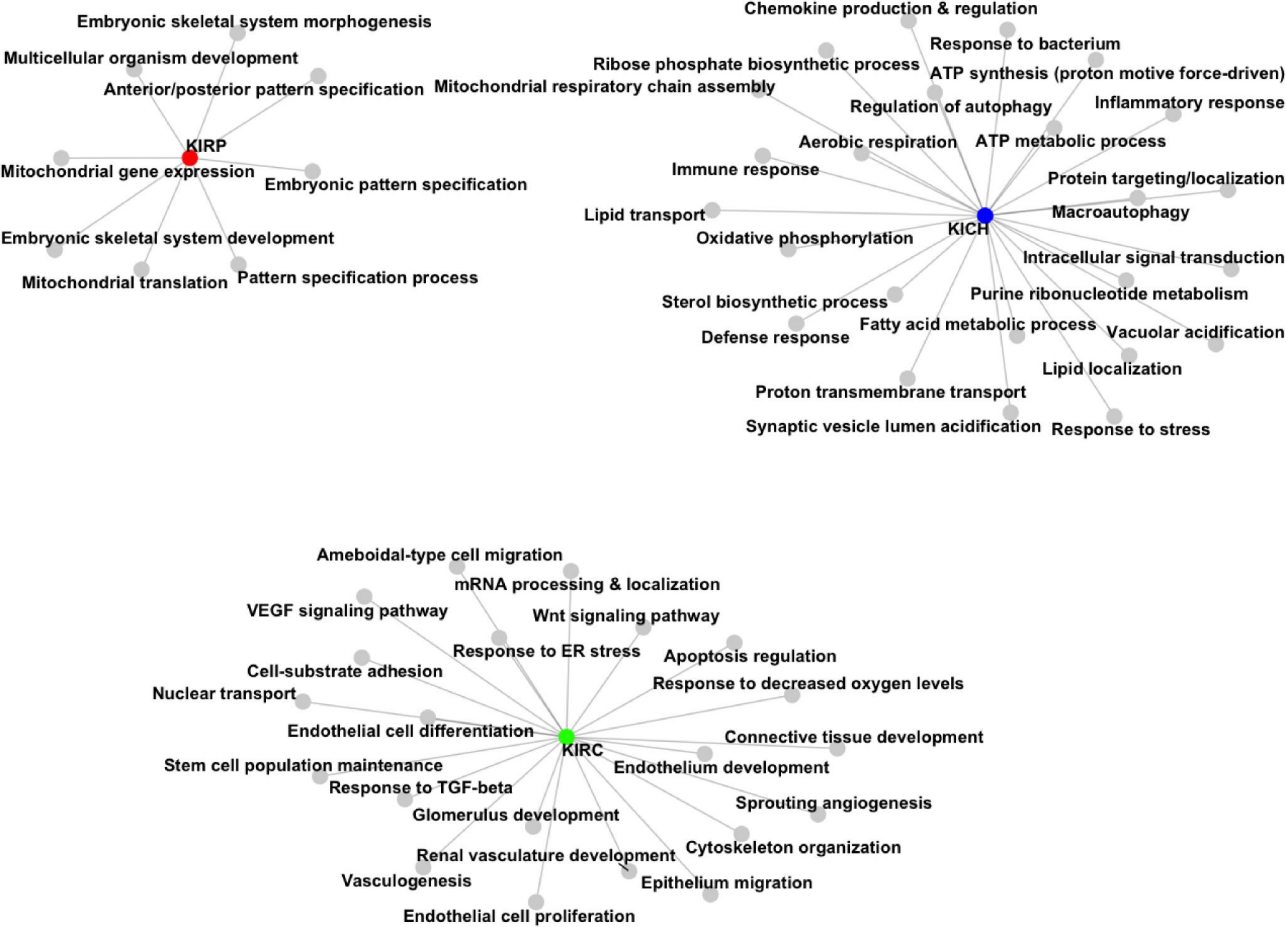
Network of enriched Gene Ontology (GO) terms by kidney cancer types. Abbreviations: KIRP, kidney renal papillary cell carcinoma; KICH, chromophobe renal cell carcinoma; KIRC, kidney renal clear cell carcinoma.

To evaluate MOBAA relative to state-of-the-art multi-omics clustering approaches, we benchmarked it against MOFA2 (Argelaguet *et al*. 2020) and iClusterPlus (Mo *et al*. 2013, 2018) using both simulated datasets with overlapping subtype structure and real data from the Cancer Genome Atlas Program (TCGA). Specifically, we included two independent cancer cohorts: the KIPAN cohort (pan-kidney cancers) and the BRCA cohort (breast invasive carcinoma). For iClusterPlus, we used *K* *=* *15* and *λ* = *0.03*, selected based on prior literature and empirical tuning to balance cluster resolution and interpretability. The relatively mild penalization (λ = 0.03) allows iClusterPlus to leverage a broader set of features, while K = 15 provides headroom to detect up to ∼15 clusters, accommodating both known subtypes and potential additional structure. For MOFA2, we used the default settings with 15 latent factors, consistent with the number of clusters used in iClusterPlus. These parameter choices were made to ensure comparability across methods and to reflect typical usage scenarios. For example, Argelaguet *et al*. (2020), recommend 10–15 factors for multi-omics integration, and iClusterPlus has been widely applied in TCGA studies with λ tuned to optimize cluster stability and biological interpretability. While different parameter settings could influence performance, we aimed to apply each method under reasonable and commonly used configurations to provide a fair and practical comparison. For the KIPAN cohort, the real-data integration included gene expression, DNA methylation and proteomics profiles from 566 participants representing three renal cancer subtypes: KIRC (kidney renal clear cell carcinoma), KIRP (kidney renal papillary cell carcinoma), and KICH (kidney chromphobe). The KIPAN cohort, encompassing diverse molecular subtypes of kidney cancer, provides an ideal dataset to demonstrate MOBAA’s ability to identify biologically distinct, multi-omic subgroups within heterogeneous disease populations. For the BRCA cohort, we used gene expression, DNA methylation and proteomics data from 179 patients with complete PAM50 subtype annotations. This cohort enabled us to further assess MOBAA’s performance in a distinct cancer type with well-established molecular subtypes, providing an additional benchmark for evaluating clustering accuracy, subtype recovery, and biological interpretability. All omics layers were standardized using z-score normalization and only patients with matched data across all three modalities were retained. This ensured that all methods were applied to the same set of omics data types measured on the same set of samples, enabling a fair and controlled comparison across methods. The methods were evaluated using subtype enrichment, cluster purity and subtype coverage, enabling a controlled comparison of their ability to recover known biological structure. MOFA2 was trained using default multi-view factorization settings, yielding 15 latent factors. Biclusters were extracted per factor by selecting the top 10% highest-loading samples and features with |z| ≥ 2, producing factor-anchored sample–feature groupings. For iClusterPlus, an integrative latent model was fit with the specified parameters across modalities. Latent embeddings were clustered using k-means, and biclusters were defined as cluster-assigned samples with features exceeding the 90th percentile of absolute β-coefficients. In contrast, MOBAA identified high-confidence biclusters without requiring cluster number specification, supporting direct comparison of specificity, sensitivity, and biological interpretability across methods. It is important to note that only MOBAA directly outputs omic features associated with each identified module, enabling downstream analyses such as inter-omic correlation and differential expression (as shown in Figs 2 and 3). In contrast, MOFA2 and iClusterPlus do not natively provide subgroup-specific feature sets. For benchmarking purposes, we derived approximate feature subsets using post hoc criteria, but these are not directly comparable to MOBAA’s module-level outputs and were not used for downstream molecular interpretation.

## 3. Results

### 3.1 Case study

To demonstrate the effectiveness of MOBAA, we applied the proposed approach to two independent multi-omics cancer cohorts from TCGA: the KIPAN and BRCA datasets. The KIPAN dataset was acquired from TCGA through Broad GDAC Firehose and the BRCA dataset was obtained using the curatedTCGAData R package.

As input from KIPAN cohort, we integrated mRNA expression (20 531 genes), protein expression (178 proteins), and DNA methylation (10 000 CpG sites) data from 566 participants in the TCGA KIPAN cohort using MOBAA. The minimum module size was set to three, corresponding to the three omics data type being analyzed, and the distance height for dynamic tree cut was set to 0.8. These parameter settings are set as the default MOBAA parameters, selected based on empirical evaluation across both the KIPAN and BRCA datasets. In both cases, these settings yielded biologically coherent modules with strong subtype enrichment, supporting their generalizability across distinct multi-omics contexts. Nevertheless, MOBAA is designed to be flexible, and users are encouraged to explore alternative parameter values to optimize module detection based on the characteristics of their specific datasets or analytical objectives. Under these settings, we identified three bicluster modules, blue, gray, and turquoise (Table 1). The blue module comprised 1 mRNA and 3 DNA methylation biclusters, the gray module comprised 13 protein, 4 mRNA, and 5 DNA methylation biclusters, and the turquoise module comprised 3 protein, 2 mRNA, and 1 DNA methylation biclusters. From each module, multi-omic relations were derived by selecting one bicluster per omic with the maximum sample overlap. Permutation-based empirical *P*-values for sample overlap among biclusters in the blue, gray, and turquoise relations were 1 × 10^−4^, .002, and 1 × 10^−4^, respectively. Notably, the overlapping samples in the blue, gray, and turquoise relations were significantly enriched for the KICH (prop.test, *P*-value < 2.2 × 10^−16^), KIRC (prop.test, *P*-value = 8.3 × 10^−5^), and KIRP (prop.test, *P*-value < 2.2 × 10^−16^) subtypes, respectively.

**Table 1:** Multi-omic relations with the constituent omic types and the number of features.

Multi-omic relations	Number of patients/samples	Omics (number of features)
*Multi-omic relations obtained from KIPAN dataset*		
Blue multi-omic relation	30	mRNA (1332); DNA methylation (1929)
Gray multi-omic relation	17	mRNA (2241); DNA methylation (1905); Protein (13)
Turquoise multi-omic relation	45	mRNA (2344); DNA methylation (3426); Protein (33)
*Multi-omic relations obtained from BRCA dataset*		
Gray multi-omic relation	16	mRNA (152); Protein (11)

Similarly, we integrated mRNA expression (12 652 genes), protein expression (216 proteins), and DNA methylation (10 000 CpG sites) data from 179 participants in the TCGA BRCA cohort using MOBAA using the same parameter setting as in KIPAN data analysis. We identified two bicluster modules, gray and turquoise (Table 1). The gray module comprised seven protein, two mRNA, and two DNA methylation biclusters, while the turquoise module comprised one protein, two mRNA, and one DNA methylation biclusters. From each module, multi-omic relations were derived by selecting one bicluster per omic with the maximum sample overlap. Permutation-based empirical *P*-values for sample overlap among biclusters in the gray and turquoise relations were 1 × 10^−4^ and .63, respectively. We proceeded with downstream analysis only for the gray module due to its statistically significant sample overlap across multi-omic biclusters. The overlapping samples in the gray relation were significantly enriched for the Basal-like subtype (prop.test, *P*-value < 2.6 × 10^−1^), highlighting MOBAA’s ability to recover biologically meaningful subgroups also in breast cancer.

Inter-omics correlation analysis in the KIPAN-based modules revealed that, in the turquoise inter-omic relation, DNA methylation is moderately, however, statistically significantly correlated with protein expression (r^2^ = 0.42, *P*-value = .005) (Fig. 2A). This finding suggests that study participants in the turquoise module show coordinated changes in DNA methylation and protein levels. Summary-level correlations among individual omic bicluster features of the gray multi-omic relation revealed weak trends between both mRNA expression and DNA methylation (*P*-value = .08) (Supplementary Fig. S2 A) and mRNA expression and protein expression (*P*-value = 0.17) (Supplementary Figure S2 B). While significant overlap of study participants among biclusters indicates multi-omic convergence, the lack of direct correlation between mRNA expression and methylation features within those biclusters likely reflects indirect regulation, different regulatory layers, or broader systemic changes not confined to simple CpG-gene pairs. Differential expression and methylation analysis comparing participants in the turquoise multi-omic relation to the rest of the cohort revealed that 89% of genes, 87% of CpG sites, and 88% of proteins were significantly differentially expressed or methylated with Bonferroni adjusted *P*-value (p.adj) < .05. The top 25 differentially expressed genes, CpG sites and proteins are presented in the Supplementary Tables S1–S3. The altered expression and methylation levels of the most significant protein and CpG site in the turquoise multi-omic relation are shown in Fig. 2B and C.

Our GO enrichment analyses revealed distinct biological programs associated with each kidney cancer subtype (Fig. 3). KIRP was uniquely characterized by enrichment of mitochondrial translation and gene expression pathways, together with embryonic developmental and pattern specification processes, suggesting a convergence of mitochondrial activity and developmental reprogramming (Supplementary Tables S4 and S5). In contrast, KICH displayed a strong metabolic signature dominated by oxidative phosphorylation and aerobic respiration, accompanied by autophagy, vacuolar acidification, and stress adaptation, highlighting its dependence on mitochondrial energy metabolism and homeostatic regulation (Supplementary Table S8). Notably, KICH also showed enrichment of immune and inflammatory response pathways, indicating a unique integration of metabolic and immune processes (Supplementary Table S9). KIRC, on the other hand, was defined by robust enrichment of angiogenesis and vascular development (including VEGF signaling), cell migration and adhesion, and cytoskeletal regulation, alongside hypoxia- and stress-responsive pathways such as TGF-β and Wnt signaling (Supplementary Table S13). These findings are consistent with the well-established role of hypoxia-driven angiogenesis in KIRC and underscore subtype-specific differences in metabolic, developmental, immune, and vascular programs that may shape tumor progression and therapeutic vulnerabilities (Davis *et al*. 2014, Nicolás-Hernández *et al*. 2025, Singh 2021). Detailed analysis of the blue and gray multi-omic relations are presented in Supplementary Sections 2 and 3.

In the BRCA-based gray module, although the gene expression and protein expression biclusters shared a common set of samples, no significant correlation was observed between the gene and protein features, suggesting broader systemic changes not confined to simple feature-feature relationships. Nevertheless, all genes and proteins within these biclusters were significantly differentially expressed when comparing the shared samples in the gray module to the remaining samples in the BRCA dataset (Supplementary Tables S14 and S15). The altered expression and protein levels of the most significant genes and protein in the gray multi-omic relation are shown in Fig. 3A and B. No biological processes were found to be significantly enriched among the differentially expressed genes in our enrichment analysis.

### 3.2 Benchmarking MOBAA against state-of-the-art methods on real multi-omics data

Because the Broad GDAC Firehose clinical files used in this study do not provide harmonized or consistently complete survival endpoints for the subset of samples with matched multi-omics data, benchmarking focused on subtype recovery, clustering performance, and biological interpretability rather than survival-based prognostic analysis. MOBAA, iClusterPlus, and MOFA2 each successfully recovered the three KIPAN subtypes but differed markedly in clustering behavior (Supplementary Table S16). MOBAA produced ultra-pure subtype-specific clusters with minimal mixing, iClusterPlus provided the most comprehensive sample coverage and revealed meaningful intra-subtype structure, while MOFA2 captured the underlying variance across subtypes through latent factors rather than discrete clusters. Together, these results illustrate that clustering precision, coverage, and interpretability vary by method, reinforcing that tool choice should align with analytical goals. In the pathway analysis, MOBAA and MOFA2 outperformed iClusterPlus in detecting subtype-relevant biological processes, identifying not only a greater number of significant biological processes but also pathways that more closely align with established mechanisms of kidney cancer subtypes (Supplementary Section 4). MOBAA, in particular, matched and in some areas exceeded MOFA2 by leveraging multi-layer methylation information—revealing additional immunoregulatory signals in chromophobe that were less apparent in the other methods. Together, MOBAA and MOFA2 captured hallmark metabolic profiles, such as oxidative metabolism in KICH and hypoxia-driven glycolysis in KIRC, suggesting that these approaches provide a more comprehensive and mechanistically grounded representation of subtype biology (Pandey *et al*. 2020). Although all three methods converge on the major metabolic distinctions across KICH, KIRC, and KIRP, MOBAA and MOFA2 deliver richer and more interpretable pathway landscapes, making them particularly valuable for downstream mechanistic exploration and hypothesis generation.

Similarly, MOBAA, iClusterPlus, and MOFA2 each successfully recovered the Basal-like subtype in the TCGA BRCA dataset but differed markedly in clustering behavior and subtype coverage (Supplementary Table S17). MOBAA produced a single ultra-pure Basal-like module with no cross-contamination, demonstrating high specificity but limited coverage across other subtypes. iClusterPlus provided the most comprehensive sample stratification, recovering multiple clusters per subtype and revealing intra-subtype heterogeneity, though with some degree of mixing, particularly for HER2-enriched and Normal-like tumors. MOFA2 captured the underlying variance across subtypes through latent factors rather than discrete clusters, identifying both pure and mixed factors associated with Basal-like and Luminal B tumors. These results reinforce that clustering precision, coverage, and interpretability vary by method, and that tool selection should be guided by the specific analytical goals of a study. Although MOBAA identified statistically significant genes in the BRCA dataset, these genes did not map to any enriched biological pathways. Consequently, pathway analysis was not performed for the other methods, as a meaningful comparison would not have been possible in the absence of a shared interpretive baseline.

We further note that Bayesian extensions such as iClusterBayes are conceptually similar to iClusterPlus, differing mainly in their inference framework (Bayesian versus penalized likelihood) (Mo *et al*. 2018). Both approaches focus on latent variable–based sample clustering and do not natively provide subgroup-specific feature identification. In contrast, MOBAA directly captures joint sample–feature structure through biclustering. As a result, inclusion of iClusterBayes would not materially change the comparative conclusions drawn here.

### 3.3 Benchmarking MOBAA against state-of-the-art methods on simulated overlapping multi-omics data

All three methods—MOBAA, MOFA2, and iClusterPlus–were able to identify subpopulation structure corresponding to the known subtypes in the simulated multi-omics data, but with notable differences in cluster purity and grouping strategy (Supplementary Section 5, Table S18). MOBAA cleanly recovered each true subtype in a single multi-omic bicluster (yielding “one cluster per subtype” with 100% subtype purity, extremely significant enrichment *P*-values). iClusterPlus also captured all subtypes but over-partitioned each subtype into multiple smaller clusters (each cluster still nearly 100% one subtype, with high significance). MOFA2 uncovered two of the three subtypes clearly (each in one latent factor with 100% purity and significant enrichment), but failed to isolate one subtype (Subtype A) as a distinct factor—that subtype’s samples were spread across several factors without a single factor exclusively enriched for it. In terms of statistical significance (prop.test) for subtype enrichment, MOBAA and iClusterPlus achieved extremely low *P*-values for all three subtypes, whereas MOFA2 achieved such significance for two subtypes (B and C) and had no factor significantly enriched for subtype A.

### 3.4 Computational performance and scalability of MOBAA

MOBAA is a modular, multi-step analysis pipeline, and its computational cost is dominated by the ensemble biclustering step applied independently to each omics layer. To assess computational feasibility, we quantified runtime and peak memory usage for the major components of the pipeline using representative multi-omics datasets of increasing dimensionality. The ensemble biclustering step (*find_ensemble_bc*), which is based on the MOSBi framework, exhibited feature-dependent scaling. For protein expression data with 178 features, ensemble biclustering completed in approximately 12 s, with a peak RAM usage of ∼3.4 GB. For DNA methylation data with 10 000 features, runtime increased to approximately 2.3 min, with peak memory usage of ∼5.6 GB. For mRNA expression data with 20 531 features, ensemble biclustering required approximately 42 min, with a similar peak memory footprint (∼5.6 GB). These results indicate that runtime scales primarily with the number of features, while memory usage remains bounded across omics types. In contrast, downstream integration steps were computationally lightweight. Construction of the bicluster presence–absence matrix and pairwise Jaccard similarity computation (*generate_jaccard_index*) completed in under one second and required less than 1 MB of additional memory. Hierarchical clustering of biclusters (*hclust_bcs*) required approximately 16 s with a peak memory usage of ∼0.2 MB. Overall, these benchmarks demonstrate that MOBAA is computationally feasible for large-scale bulk multi-omics datasets comprising tens of thousands of features per modality on standard high-memory workstations. While MOBAA can, in principle, be applied to single-cell multi-omics data, practical application in that setting may require feature filtering, aggregation, or dimensionality reduction prior to ensemble biclustering to ensure scalability. Given the extreme sparsity and dropout rates characteristic of single-cell transcriptomics, future extensions of MOBAA may benefit from incorporating more robust strategies such as hierarchical feature detection or graph-based imputation to better preserve rare subpopulation signals during preprocessing.

## 4. Conclusion

MOBAA offers a robust and scalable framework for the identification of biologically and clinically meaningful subgroups through the integrative analysis of multi-omic data. A key novelty of MOBAA lies in its fully distribution-free design and its ability to directly output subgroup-specific multi-omic features, in contrast to latent-factor-based approaches that require indirect interpretation. Its application successfully revealed distinct molecular signatures aligned with known disease subtypes, demonstrating its utility in extracting clinically relevant insights from complex, heterogeneous, multi-layered datasets. By leveraging a module-based strategy based on sample overlap rather than parametric modeling assumptions, MOBAA is broadly applicable to diverse multi-omics settings and can, in principle, be extended to other data modalities, including single-cell multi-omics, where heterogeneity and sparsity are prominent. While multi-omics integration can enhance subtype discovery, adding more omics layers does not always improve performance. Integration is most effective when the selected layers capture complementary biological information, such as gene expression, genomic alterations (mutations and copy number), and epigenetic regulation (DNA methylation or proteomics). Noisy or highly redundant layers may reduce clustering accuracy. MOBAA provides a flexible framework that allows users to integrate omics layers based on biological relevance and data quality and empirically evaluate their contribution to subtype separation. Overall, MOBAA enables the discovery of coordinated molecular patterns across multiple omic layers, supporting advances in precision medicine and biomarker discovery.

## Supplementary Material

vbag156_Supplementary_Data

## Data Availability

The MOBAA R package, along with use-case scenarios and comprehensive documentation, is available at https://github.com/pmishra912/MOBAA. The software is open-source and distributed under the MIT License. The KIPAN omics dataset was obtained from TCGA through the Broad GDAC Firehouse (https://gdac.broadinstitute.org/).
