# Supplementary material for: Multi-Omic Bicluster Association Analysis (MOBAA)—a tool for identifying population subgroups with distinct multi-omics molecular profiles
Source: Bioinform Adv. 2026 Jun 3;6(1):vbag156. doi: 10.1093/bioadv/vbag156 (PMC13275120; doi:10.1093/bioadv/vbag156)
Supplement: vbag156_Supplementary_Data [file vbag156_supplementary_data.docx]

**Supplementary materials**

**Multi-Omic Bicluster Association Analysis (MOBAA)–A tool for identifying population subgroups with distinct multi-omics molecular profile**

Binisha H. Mishra^1,2,3,4*^, Pashupati P. Mishra^1,2,3,4*#^

**Affiliations:**

^1^Department of Clinical Chemistry, Faculty of Medicine and Health Technology, Tampere University, Tampere, Finland

^2^Finnish Cardiovascular Research Center Tampere, Faculty of Medicine and Health Technology, Tampere University, Tampere, Finland

^3^Department of Clinical Chemistry, Fimlab Laboratories, Tampere, Finland

^4^Tampere University Hospital, Wellbeing Services county of Pirkanmaa, Tampere, Finland

* Equal contribution

# Corresponding author: pashupati.mishra@tuni.fi

**Results from KIPAN data analysis**

1. **Turquoise multi-omic relation**

**Table S1**. Top 25 differentially expressed genes from the turquoise multi-omic relation, ranked by statistical significance. The table includes the feature name, test statistic estimate, p-value, and direction of change compared to unrelated participants.

| **Feature** | **Test estimate** | **P value** | **Direction** | **P.adjust** |
| --- | --- | --- | --- | --- |
| GPS1\|2873 | -0.60 | 9.73e-35 | upregulated | 2.28e-31 |
| ANXA11\|311 | -1.20 | 3.10e-34 | upregulated | 7.26e-31 |
| SPAG7\|9552 | -0.32 | 9.20e-34 | upregulated | 2.16e-30 |
| PFN1\|5216 | -2.36 | 1.71e-33 | upregulated | 4.00e-30 |
| UBAC2\|337867 | -0.43 | 2.84e-33 | upregulated | 6.65e-30 |
| HMGB3\|3149 | -0.29 | 3.38e-33 | upregulated | 7.93e-30 |
| HNF1B\|6928 | -1.76 | 4.13e-33 | upregulated | 9.69e-30 |
| TMUB2\|79089 | -0.24 | 3.15e-32 | upregulated | 7.37e-29 |
| TMEM115\|11070 | -0.25 | 3.67e-32 | upregulated | 8.61e-29 |
| UBE2I\|7329 | -0.22 | 4.00e-32 | upregulated | 9.38e-29 |
| PLA2G16\|11145 | -0.90 | 4.22e-32 | upregulated | 9.89e-29 |
| TAF6\|6878 | -0.56 | 4.49e-32 | upregulated | 1.05e-28 |
| VPS25\|84313 | -0.39 | 5.73e-32 | upregulated | 1.34e-28 |
| ELAC2\|60528 | -0.42 | 6.47e-32 | upregulated | 1.52e-28 |
| SIRT3\|23410 | -0.19 | 6.98e-32 | upregulated | 1.64e-28 |
| C2orf28\|51374 | -0.39 | 7.23e-32 | upregulated | 1.70e-28 |
| JMJD8\|339123 | -0.55 | 8.57e-32 | upregulated | 2.01e-28 |
| MRPL38\|64978 | -0.39 | 9.91e-32 | upregulated | 2.32e-28 |
| MEN1\|4221 | -0.10 | 1.46e-31 | upregulated | 3.42e-28 |
| WIPI2\|26100 | -0.49 | 1.50e-31 | upregulated | 3.51e-28 |
| SAP30BP\|29115 | -0.19 | 2.95e-31 | upregulated | 6.91e-28 |
| SSH3\|54961 | -0.29 | 3.20e-31 | upregulated | 7.50e-28 |
| STXBP2\|6813 | -0.24 | 7.07e-31 | upregulated | 1.66e-27 |
| MAD1L1\|8379 | -0.21 | 7.64e-31 | upregulated | 1.79e-27 |
| P4HTM\|54681 | -0.27 | 9.41e-31 | upregulated | 2.21e-27 |

**Table S2**. Top 25 differentially methylated CpG sites from the turquoise multi-omic relation, ranked by statistical significance. The table includes the feature name, test statistic estimate, p-value, and direction of change compared to unrelated participants.

| **Feature** | **Test estimate** | **P_value** | **Direction** | **p.adjust** |
| --- | --- | --- | --- | --- |
| cg27481555 | -0.44 | 2.27e-164 | upregulated | 7.79e-161 |
| cg16586594 | -0.41 | 1.49e-149 | upregulated | 5.09e-146 |
| cg04334243 | -0.40 | 2.86e-148 | upregulated | 9.80e-145 |
| cg22084642 | -0.45 | 4.96e-148 | upregulated | 1.70e-144 |
| cg25150953 | -0.42 | 1.16e-143 | upregulated | 3.96e-140 |
| cg06012347 | -0.34 | 8.48e-142 | upregulated | 2.90e-138 |
| cg23605961 | -0.39 | 1.12e-138 | upregulated | 3.83e-135 |
| cg03763518 | -0.42 | 6.50e-138 | upregulated | 2.23e-134 |
| cg05915866 | -0.45 | 3.41e-137 | upregulated | 1.17e-133 |
| cg06939852 | -0.34 | 4.05e-137 | upregulated | 1.39e-133 |
| cg04917446 | -0.43 | 1.25e-134 | upregulated | 4.28e-131 |
| cg00088797 | -0.32 | 2.98e-134 | upregulated | 1.02e-130 |
| cg03308399 | -0.32 | 3.18e-133 | upregulated | 1.09e-129 |
| cg08834902 | -0.33 | 2.73e-132 | upregulated | 9.36e-129 |
| cg23918296 | -0.36 | 4.47e-132 | upregulated | 1.53e-128 |
| cg00397479 | -0.47 | 6.58e-132 | upregulated | 2.25e-128 |
| cg06200244 | -0.39 | 3.41e-131 | upregulated | 1.17e-127 |
| cg12902206 | -0.44 | 3.56e-129 | upregulated | 1.22e-125 |
| cg01964852 | -0.38 | 8.56e-129 | upregulated | 2.93e-125 |
| cg00220102 | -0.33 | 2.81e-128 | upregulated | 9.64e-125 |
| cg20442697 | -0.33 | 3.54e-128 | upregulated | 1.21e-124 |
| cg10313047 | -0.34 | 1.63e-127 | upregulated | 5.59e-124 |
| cg02484210 | -0.35 | 2.41e-127 | upregulated | 8.25e-124 |
| cg18971175 | -0.32 | 2.76e-127 | upregulated | 9.45e-124 |
| cg14483162 | -0.37 | 3.31e-127 | upregulated | 1.14e-123 |

**Table S3**. List of differentially expressed proteins from the turquoise multi-omic relation, ranked by statistical significance. The table includes the feature name, test statistic estimate, p-value, and direction of change compared to unrelated participants.

| **Feature** | **Test_estimate** | **P_value** | **Direction** | **p.adjust** |
| --- | --- | --- | --- | --- |
| GSK3-alpha-beta_pS21_S9 | -1.29 | 6.24e-40 | upregulated | 2.06e-38 |
| PKC-pan_BetaII_pS660 | -1.27 | 6.05e-37 | upregulated | 2.00e-35 |
| beta-Catenin | -1.21 | 2.09e-33 | upregulated | 6.88e-32 |
| ATM | -1.35 | 1.81e-32 | upregulated | 5.99e-31 |
| Akt | -1.07 | 1.14e-30 | upregulated | 3.76e-29 |
| RBM15 | -1.26 | 1.41e-30 | upregulated | 4.66e-29 |
| mTOR | -0.90 | 3.55e-29 | upregulated | 1.17e-27 |
| GSK3_pS9 | -1.29 | 3.68e-29 | upregulated | 1.21e-27 |
| AMPK_alpha | -0.89 | 2.09e-26 | upregulated | 6.88e-25 |
| HER2 | -1.14 | 6.26e-24 | upregulated | 2.07e-22 |
| p38_pT180_Y182 | -1.67 | 1.12e-23 | upregulated | 3.68e-22 |
| PDK1_pS241 | -0.62 | 4.66e-22 | upregulated | 1.54e-20 |
| Tuberin | -0.84 | 3.71e-18 | upregulated | 1.22e-16 |
| Bad_pS112 | -0.53 | 7.17e-17 | upregulated | 2.37e-15 |
| B-Raf | -0.84 | 8.96e-17 | upregulated | 2.96e-15 |
| TSC1 | -0.58 | 1.73e-16 | upregulated | 5.72e-15 |
| mTOR_pS2448 | -0.50 | 7.11e-16 | upregulated | 2.34e-14 |
| Src_pY527 | -1.38 | 2.50e-15 | upregulated | 8.25e-14 |
| AR | -0.77 | 3.22e-14 | upregulated | 1.06e-12 |
| PKC-alpha_pS657 | -1.02 | 1.31e-13 | upregulated | 4.34e-12 |
| GSK3-alpha-beta | -0.36 | 1.69e-11 | upregulated | 5.57e-10 |
| PTEN | -0.62 | 1.03e-10 | upregulated | 3.40e-09 |
| p70S6K | -0.38 | 1.06e-10 | upregulated | 3.50e-09 |
| NF2 | -0.69 | 2.66e-10 | upregulated | 8.78e-09 |
| Ku80 | -0.44 | 1.23e-09 | upregulated | 4.06e-08 |

**Table S4**. Gene Ontology (GO) biological processes significantly overrepresented among genes in the turquoise multi-omic relation.

| **ID** | **Description** | **P-value** | **P.adjust** |
| --- | --- | --- | --- |
| GO:0032543 | Mitochondrial translation | 4.2e-08 | 0.0002 |
| GO:0140053 | Mitochondrial gene expression | 1.3e-07 | 0.0006 |

**Table S5**. Gene Ontology (GO) biological processes significantly overrepresented among CpG sites in the turquoise multi-omic relation.

| **ID** | **Description** | **P-value** | **FDR** |
| --- | --- | --- | --- |
| GO:0009952 | Anterior/posterior pattern specification | 2.1e-07 | 0.005 |
| GO:0048706 | Embryonic skeletal system development | 7.7e-07 | 0.009 |
| GO:0007275 | Multicellular organism development | 2.9e-06 | 0.02 |
| GO:0048704 | Embryonic skeletal system morphogenesis | 2.3e-06 | 0.02 |
| GO:0009880 | Embryonic pattern specification | 8.3e-06 | 0.04 |
| GO:0007389 | Pattern specification process | 9.9e-06 | 0.04 |

1. **Blue multi-omic relation**

***Summary-level correlation among individual omic bicluster features*** Summary-level correlations among individual omic bicluster features revealed no association between mRNA expression and DNA methylation. This suggests that, although biclusters from different omics share a significant number of participants, indicating multi-omic convergence, the absence of direct correlation between expression and methylation features likely reflects indirect regulatory mechanisms, distinct regulatory layers, or broader systemic changes beyond simple CpG–gene pair relationships.

***Differential expression and methylation analysis*** comparing participants in the blue multi-omic relation to the rest of the cohort revealed that 95% of genes and 90% of CpG sites were significantly differentially expressed or methylated with Bonferroni adjusted p-value (p.adj) < 0.05. The top 25 differentially expressed genes and CpG sites are presented in Tables S6-S7. The altered methylation and expression levels of the most significant CpG site and gene in the blue multi-omic relation are shown in Figure S1.

**Table S6**. Top 25 differentially expressed genes from the blue multi-omic relation, ranked by statistical significance. The table includes the feature name, test statistic estimate, p-value, and direction of change compared to unrelated participants.

| **Feature** | **Test estimate** | **P_value** | **Direction** | **p.adjust** |
| --- | --- | --- | --- | --- |
| FOXI1\|2299 | -4.76 | 2.46e-23 | upregulated | 3.27e-20 |
| SLC25A3\|5250 | -3.87 | 4.24e-21 | upregulated | 5.65e-18 |
| ISCA2\|122961 | -0.18 | 1.22e-20 | upregulated | 1.62e-17 |
| GOT2\|2806 | -1.16 | 1.94e-20 | upregulated | 2.58e-17 |
| C14orf159\|80017 | -1.14 | 2.54e-20 | upregulated | 3.38e-17 |
| TSPAN31\|6302 | -0.32 | 2.76e-20 | upregulated | 3.68e-17 |
| ATP6V0C\|527 | -5.51 | 3.04e-20 | upregulated | 4.05e-17 |
| SLBP\|7884 | -0.37 | 3.51e-20 | upregulated | 4.67e-17 |
| CLPB\|81570 | -0.21 | 6.42e-20 | upregulated | 8.55e-17 |
| NDRG2\|57447 | -1.97 | 9.03e-20 | upregulated | 1.20e-16 |
| GAK\|2580 | -1.06 | 1.80e-19 | upregulated | 2.39e-16 |
| MAEA\|10296 | -0.59 | 1.83e-19 | upregulated | 2.44e-16 |
| TBC1D1\|23216 | -4.64 | 2.79e-19 | upregulated | 3.72e-16 |
| MAPKSP1\|8649 | -0.33 | 3.69e-19 | upregulated | 4.92e-16 |
| WDR20\|91833 | -0.10 | 5.08e-19 | upregulated | 6.76e-16 |
| OGDHL\|55753 | -5.83 | 8.65e-19 | upregulated | 1.15e-15 |
| GLTP\|51228 | -2.07 | 9.58e-19 | upregulated | 1.28e-15 |
| ISCU\|23479 | -2.21 | 2.54e-18 | upregulated | 3.38e-15 |
| SCNN1A\|6337 | -6.57 | 4.78e-18 | upregulated | 6.37e-15 |
| ATP6V1D\|51382 | -1.34 | 7.61e-18 | upregulated | 1.01e-14 |
| TMED1\|11018 | -0.39 | 1.04e-17 | upregulated | 1.39e-14 |
| C20orf3\|57136 | -0.83 | 1.10e-17 | upregulated | 1.46e-14 |
| VTI1B\|10490 | -0.67 | 1.11e-17 | upregulated | 1.48e-14 |
| NDUFA9\|4704 | -0.62 | 1.44e-17 | upregulated | 1.92e-14 |
| TBC1D14\|57533 | -9.65 | 1.47e-17 | upregulated | 1.96e-14 |

**Table S7**. Top 25 differentially expressed genes from the blue multi-omic relation, ranked by statistical significance. The table includes the feature name, test statistic estimate, p-value, and direction of change compared to unrelated participants.

| **Feature** | **Test estimate** | **P_value** | **Direction** | **p.adjust** |
| --- | --- | --- | --- | --- |
| cg02748089 | -0.60 | 3.01e-244 | upregulated | 5.81e-241 |
| cg25296103 | -0.55 | 1.71e-202 | upregulated | 3.29e-199 |
| cg08565003 | -0.47 | 4.25e-194 | upregulated | 8.19e-191 |
| cg11254532 | -0.46 | 9.77e-193 | upregulated | 1.88e-189 |
| cg14050824 | -0.46 | 1.29e-186 | upregulated | 2.50e-183 |
| cg01824603 | -0.41 | 2.05e-178 | upregulated | 3.95e-175 |
| cg04482075 | -0.42 | 3.99e-178 | upregulated | 7.71e-175 |
| cg17942750 | -0.45 | 1.60e-171 | upregulated | 3.08e-168 |
| cg15725733 | -0.37 | 7.25e-171 | upregulated | 1.40e-167 |
| cg01295782 | -0.47 | 6.35e-167 | upregulated | 1.22e-163 |
| cg21201401 | -0.50 | 7.65e-166 | upregulated | 1.48e-162 |
| cg13045134 | -0.51 | 5.52e-160 | upregulated | 1.06e-156 |
| cg16233472 | -0.45 | 6.22e-160 | upregulated | 1.20e-156 |
| cg23386212 | -0.38 | 2.94e-157 | upregulated | 5.68e-154 |
| cg00446123 | -0.46 | 3.12e-154 | upregulated | 6.02e-151 |
| cg12402318 | -0.41 | 6.71e-151 | upregulated | 1.30e-147 |
| cg00651016 | -0.40 | 1.55e-150 | upregulated | 2.99e-147 |
| cg01885839 | -0.40 | 4.23e-150 | upregulated | 8.16e-147 |
| cg15997512 | -0.37 | 1.30e-149 | upregulated | 2.50e-146 |
| cg26936171 | -0.36 | 4.82e-149 | upregulated | 9.31e-146 |
| cg14459021 | -0.39 | 9.44e-149 | upregulated | 1.82e-145 |
| cg19850370 | -0.43 | 1.31e-148 | upregulated | 2.52e-145 |
| cg03332897 | -0.41 | 2.79e-147 | upregulated | 5.38e-144 |
| cg16547869 | -0.37 | 2.90e-146 | upregulated | 5.60e-143 |
| cg20024687 | -0.33 | 9.20e-146 | upregulated | 1.77e-142 |

**
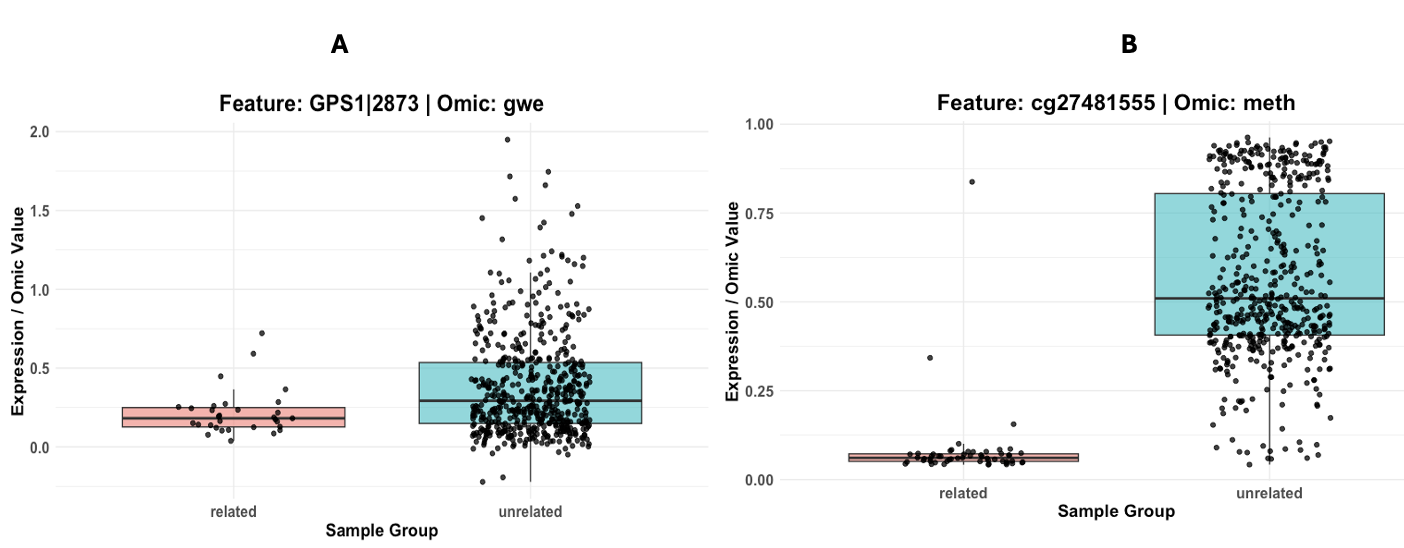
**

**Figure S1**. Boxplots showing differences in expression and methylation levels of the top-ranked genen and CpG site between participants within the blue multi-omic relation (‘related’) and those outside the relation in the rest of the cohort (‘unrelated’).

***Pathway enrichment analysis*** of the omic features involved in the blue inter-omic relations are presented in Tables S8-S9.

**Table S8**. Gene Ontology (GO) biological processes significantly overrepresented among genes in the blue multi-omic relation.

| **ID** | **Description** | **P-value** | **P.adjust** |
| --- | --- | --- | --- |
| GO:0009060 | aerobic respiration | 1.2e-29 | 6.3e-26 |
| GO:0006119 | oxidative phosphorylation | 2.7e-26 | 1.4e-22 |
| GO:1902600 | proton transmembrane transport | 5.1e-19 | 2.7e-15 |
| GO:0015986 | proton motive force-driven ATP synthesis | 2.2e-17 | 1.1e-13 |
| GO:0046390 | ribose phosphate biosynthetic process | 2.3e-13 | 1.2e-09 |
| GO:0046034 | ATP metabolic process | 1.4e-12 | 7.3e-09 |
| GO:0009150 | purine ribonucleotide metabolic process | 7.8e-12 | 4.1e-08 |
| GO:0010506 | regulation of autophagy | 1.7e-10 | 9.0e-07 |
| GO:0033108 | mitochondrial respiratory chain complex assembly | 2.2e-10 | 1.1e-06 |
| GO:0097401 | synaptic vesicle lumen acidification | 7.3e-10 | 3.9e-06 |
| GO:0016236 | macroautophagy | 2.3e-09 | 1.2e-05 |
| GO:0007035 | vacuolar acidification | 3.0e-09 | 1.6e-05 |
| GO:0016188 | synaptic vesicle maturation | 9.1e-09 | 4.8e-05 |
| GO:0006885 | regulation of pH | 9.1e-09 | 5.1e-05 |
| GO:0010821 | regulation of mitochondrion organization | 7.3e-08 | 3.9e-04 |
| GO:0006605 | protein targeting | 9.8e-08 | 5.2e-04 |
| GO:0010876 | lipid localization | 3.6e-07 | 1.9e-03 |
| GO:0006839 | mitochondrial transport | 6.8e-07 | 3.6e-03 |
| GO:0070585 | protein localization to mitochondrion | 1.2e-06 | 6.2e-03 |
| GO:0007006 | mitochondrial membrane organization | 1.6e-06 | 8.2e-03 |
| GO:0006066 | alcohol metabolic process | 2.3e-06 | 1.2e-02 |
| GO:0016126 | sterol biosynthetic process | 3.1e-06 | 1.6e-02 |
| GO:0006631 | fatty acid metabolic process | 3.5e-06 | 1.8e-02 |
| GO:0048193 | Golgi vesicle transport | 4.2e-06 | 2.2e-02 |
| GO:0006869 | lipid transport | 4.5e-06 | 2.4e-02 |
| GO:0051223 | regulation of protein transport | 4.8e-06 | 2.5e-02 |
| GO:1903829 | positive regulation of protein localization | 7.3e-06 | 3.8e-02 |
| GO:0072659 | protein localization to plasma membrane | 7.5e-06 | 3.9e-02 |

**Table S9**. Gene Ontology (GO) biological processes significantly overrepresented among CpG sites in the blue multi-omic relation.

| **ID** | **Description** | **P-value** | **FDR** |
| --- | --- | --- | --- |
| GO:0006952 | defense response | 1.1e-07 | 0.001 |
| GO:0006954 | inflammatory response | 1.2e-07 | 0.001 |
| GO:0044281 | small molecule metabolic process | 3.5e-07 | 0.002 |
| GO:0009617 | response to bacterium | 7.4e-07 | 0.003 |
| GO:0006955 | immune response | 2.9e-06 | 0.01 |
| GO:0019752 | carboxylic acid metabolic process | 7.9e-06 | 0.02 |
| GO:0006082 | organic acid metabolic process | 1.9e-05 | 0.04 |
| GO:0006950 | response to stress | 1.8e-05 | 0.04 |
| GO:0032496 | response to lipopolysaccharide | 2.0e-05 | 0.04 |
| GO:0043436 | oxoacid metabolic process | 1.6e-05 | 0.04 |
| GO:0002237 | response to molecule of bacterial origin | 2.3e-05 | 0.04 |
| GO:0043207 | response to external biotic stimulus | 2.7e-05 | 0.04 |
| GO:0051707 | response to other organism | 2.6e-05 | 0.04 |
| GO:0071219 | cellular response to molecule of bacterial origin | 2.6e-05 | 0.04 |
| GO:0044419 | biological process involved in interspecies interaction between organisms | 3.2e-05 | 0.04 |
| GO:0032642 | regulation of chemokine production | 3.7e-05 | 0.04 |
| GO:0032602 | chemokine production | 3.9e-05 | 0.04 |
| GO:0071222 | cellular response to lipopolysaccharide | 4.2e-05 | 0.04 |
| GO:0035556 | intracellular signal transduction | 4.9e-05 | 0.047 |
| GO:0050896 | response to stimulus | 5.0e-05 | 0.047 |

1. **Grey multi-omic relation**

**
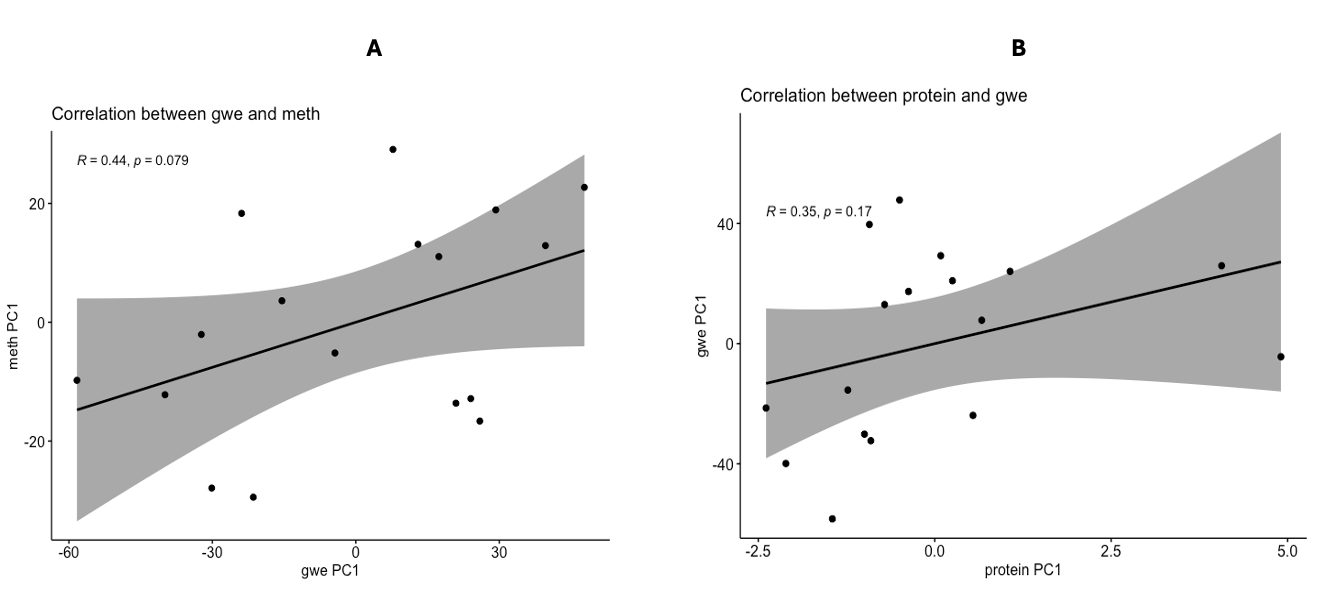
**

**Figure S2. A.** Correlation between eigenfeatures (first principal component) of DNA methylation and mRNA expression in the grey multi-omic relations. **B**. Correlation between eigenfeatures of mRNA expression and protein expression in the grey multi-omic relations.

***Differential expression and methylation analysis*** comparing participants in the grey multi-omic relation to the rest of the cohort revealed that 88% of genes, 88% of CpG sites and 69% of the proteins were significantly differentially expressed or methylated with p.adj < 0.05. The top 25 differentially expressed genes and CpG sites and the list of differentially expressed proteins are presented in Tables S10-S12. The altered methylation and expression levels of the most significant CpG site, gene and protein in the grey multi-omic relation are shown in Figure S3.

**Table S10**. Top 25 differentially expressed genes from the grey multi-omic relation, ranked by statistical significance. The table includes the feature name, test statistic estimate, p-value, and direction of change compared to unrelated participants.

| **feature** | **test_estimate** | **p_value** | **direction** | **p.adjust** |
| --- | --- | --- | --- | --- |
| PEA15\|8682 | -0.97 | 2.89e-55 | upregulated | 6.48e-52 |
| NRP1\|8829 | -2.51 | 5.18e-53 | upregulated | 1.16e-49 |
| GIMAP4\|55303 | -0.43 | 1.19e-52 | upregulated | 2.67e-49 |
| A2M\|2 | -9.32 | 4.83e-52 | upregulated | 1.08e-48 |
| DPYSL2\|1808 | -0.82 | 8.91e-52 | upregulated | 2.00e-48 |
| EHD2\|30846 | -1.61 | 9.21e-52 | upregulated | 2.06e-48 |
| VIM\|7431 | -14.03 | 1.78e-51 | upregulated | 3.98e-48 |
| STOM\|2040 | -1.45 | 1.59e-50 | upregulated | 3.56e-47 |
| GPR4\|2828 | -0.39 | 5.94e-50 | upregulated | 1.33e-46 |
| JAG1\|182 | -1.14 | 6.88e-50 | upregulated | 1.54e-46 |
| FLT1\|2321 | -4.32 | 1.07e-49 | upregulated | 2.40e-46 |
| LDB2\|9079 | -0.48 | 2.49e-49 | upregulated | 5.57e-46 |
| MSN\|4478 | -2.49 | 4.24e-49 | upregulated | 9.50e-46 |
| UBA2\|10054 | -0.22 | 1.49e-48 | upregulated | 3.33e-45 |
| MEF2C\|4208 | -0.58 | 4.74e-48 | upregulated | 1.06e-44 |
| FLI1\|2313 | -0.23 | 1.10e-47 | upregulated | 2.46e-44 |
| ARHGDIB\|397 | -1.15 | 1.25e-47 | upregulated | 2.81e-44 |
| CYYR1\|116159 | -0.36 | 1.54e-46 | upregulated | 3.45e-43 |
| INSR\|3643 | -2.34 | 1.19e-45 | upregulated | 2.66e-42 |
| SPARC\|6678 | -15.83 | 1.81e-45 | upregulated | 4.07e-42 |
| ETS1\|2113 | -2.12 | 5.24e-45 | upregulated | 1.18e-41 |
| PRSS23\|11098 | -1.33 | 1.32e-44 | upregulated | 2.96e-41 |
| EPAS1\|2034 | -5.68 | 2.81e-44 | upregulated | 6.31e-41 |
| ESAM\|90952 | -0.81 | 4.58e-44 | upregulated | 1.03e-40 |
| MMRN2\|79812 | -0.83 | 1.09e-43 | upregulated | 2.45e-40 |

**Table S11**. Top 25 differentially expressed genes from the grey multi-omic relation, ranked by statistical significance. The table includes the feature name, test statistic estimate, p-value, and direction of change compared to unrelated participants.

| **feature** | **test_estimate** | **p_value** | **direction** | **p.adjust** |
| --- | --- | --- | --- | --- |
| cg14765933 | -0.40 | 9.78e-103 | upregulated | 1.86e-99 |
| cg02906741 | -0.30 | 1.58e-95 | upregulated | 3.00e-92 |
| cg16479247 | -0.27 | 7.05e-84 | upregulated | 1.34e-80 |
| cg18236877 | -0.41 | 2.98e-83 | upregulated | 5.67e-80 |
| cg10050410 | -0.31 | 2.73e-81 | upregulated | 5.21e-78 |
| cg20187173 | -0.31 | 9.61e-81 | upregulated | 1.83e-77 |
| cg04511534 | -0.28 | 3.72e-79 | upregulated | 7.08e-76 |
| cg08316831 | -0.30 | 1.12e-78 | upregulated | 2.14e-75 |
| cg13776620 | -0.28 | 5.58e-78 | upregulated | 1.06e-74 |
| cg02691393 | -0.30 | 5.71e-78 | upregulated | 1.09e-74 |
| cg14633742 | -0.36 | 1.31e-76 | upregulated | 2.49e-73 |
| cg14155027 | -0.30 | 1.12e-75 | upregulated | 2.13e-72 |
| cg19687152 | -0.29 | 1.30e-75 | upregulated | 2.49e-72 |
| cg04388901 | -0.36 | 3.22e-74 | upregulated | 6.13e-71 |
| cg13144143 | -0.30 | 5.32e-74 | upregulated | 1.01e-70 |
| cg10289744 | -0.35 | 6.24e-74 | upregulated | 1.19e-70 |
| cg14398860 | -0.35 | 1.01e-73 | upregulated | 1.92e-70 |
| cg15488978 | -0.30 | 1.72e-73 | upregulated | 3.27e-70 |
| cg04896832 | -0.38 | 5.10e-73 | upregulated | 9.71e-70 |
| cg08658787 | -0.30 | 8.19e-73 | upregulated | 1.56e-69 |
| cg00283857 | -0.31 | 1.41e-72 | upregulated | 2.69e-69 |
| cg01397065 | -0.27 | 2.67e-72 | upregulated | 5.09e-69 |
| cg05956452 | -0.32 | 3.33e-72 | upregulated | 6.35e-69 |
| cg19254118 | -0.38 | 6.83e-72 | upregulated | 1.30e-68 |
| cg01426041 | -0.28 | 2.41e-71 | upregulated | 4.59e-68 |

**Table S12**. List of differentially expressed proteins from the grey multi-omic relation, ranked by statistical significance. The table includes the feature name, test statistic estimate, p-value, and direction of change compared to unrelated participants.

| **feature** | **test_estimate** | **p_value** | **direction** | **p.adjust** |
| --- | --- | --- | --- | --- |
| VEGFR2 | -1.59 | 1.30e-33 | upregulated | 1.69e-32 |
| TSC1 | -0.67 | 9.89e-21 | upregulated | 1.29e-19 |
| beta-Catenin | -0.82 | 1.62e-16 | upregulated | 2.11e-15 |
| GSK3-alpha-beta | -0.48 | 1.79e-15 | upregulated | 2.33e-14 |
| Akt | -0.67 | 3.64e-15 | upregulated | 4.73e-14 |
| Claudin-7 | -1.39 | 3.06e-12 | upregulated | 3.98e-11 |
| HER2 | -0.68 | 8.36e-12 | upregulated | 1.09e-10 |
| C-Raf | -0.33 | 5.81e-10 | upregulated | 7.55e-09 |
| E-Cadherin | -0.76 | 2.06e-06 | upregulated | 2.68e-05 |
| YAP_pS127 | -0.28 | 2.43e-02 | upregulated | 3.16e-01 |
| Rictor | -0.19 | 1.13e-01 | upregulated | 1.00e+00 |
| Rab25 | -0.11 | 2.86e-01 | upregulated | 1.00e+00 |
| HER2_pY1248 | -0.05 | 4.25e-01 | upregulated | 1.00e+00 |


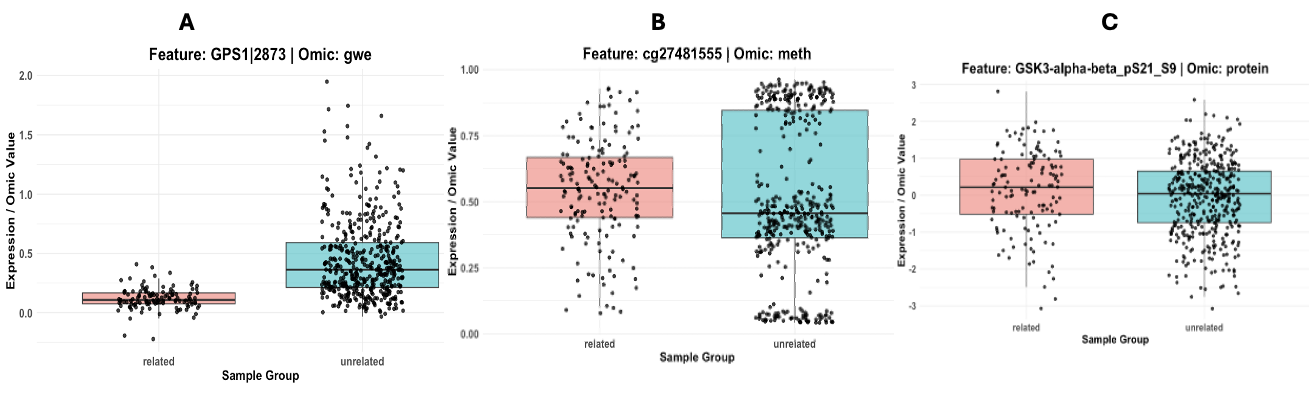


**Figure S3**. Boxplots showing differences in top-ranked: **A)** mRNA expression, **B)** methylation levels, and **C)** protein expression levels of the participants within the grey multi-omic relation between (‘related’) and those outside the relation in the rest of the cohort (‘unrelated’).

***Pathway enrichment analysis*** of the omic features involved in the grey inter-omic relations are presented in Tables S8-S9.

**Table S13**. Gene Ontology (GO) biological processes significantly overrepresented among genes in the grey multi-omic relation

| **ID** | **Description** | **P-value** | **p.adjust** |
| --- | --- | --- | --- |
| GO:0001667 | ameboidal-type cell migration | 4.8e-23 | 2.8e-19 |
| GO:0007264 | small GTPase mediated signal transduction | 1.7e-22 | 1.0e-18 |
| GO:0090132 | epithelium migration | 1.2e-19 | 6.8e-16 |
| GO:0071559 | response to transforming growth factor beta | 1.9e-18 | 1.1e-14 |
| GO:0003158 | endothelium development | 1.3e-17 | 7.6e-14 |
| GO:0071560 | cellular response to transforming growth factor beta stimulus | 1.8e-17 | 1.0e-13 |
| GO:0031589 | cell-substrate adhesion | 4.3e-17 | 2.5e-13 |
| GO:1901342 | regulation of vasculature development | 3.1e-16 | 1.8e-12 |
| GO:0045446 | endothelial cell differentiation | 3.0e-15 | 1.8e-11 |
| GO:0002040 | sprouting angiogenesis | 4.0e-15 | 2.4e-11 |
| GO:0031400 | negative regulation of protein modification process | 6.8e-15 | 4.0e-11 |
| GO:0010810 | regulation of cell-substrate adhesion | 1.3e-14 | 7.5e-11 |
| GO:0010632 | regulation of epithelial cell migration | 1.8e-14 | 1.1e-10 |
| GO:0045936 | negative regulation of phosphate metabolic process | 3.9e-13 | 2.3e-09 |
| GO:0001935 | endothelial cell proliferation | 8.3e-13 | 4.9e-0 |
| GO:0040013 | negative regulation of locomotion | 1.4e-12 | 8.2e-09 |
| GO:0007178 | transmembrane receptor protein serine/threonine kinase signaling pathway | 9.2e-12 | 5.4e-08 |
| GO:0003170 | heart valve development | 1.9e-11 | 1.1e-07 |
| GO:0001570 | vasculogenesis | 2.0e-11 | 1.2e-07 |
| GO:0150115 | cell-substrate junction organization | 2.2e-11 | 1.3e-07 |
| GO:0003007 | heart morphogenesis | 3.3e-11 | 2.0e-07 |
| GO:0036293 | response to decreased oxygen levels | 6.2e-11 | 3.6e-07 |
| GO:0006417 | regulation of translation | 7.5e-11 | 4.4e-07 |
| GO:0007015 | actin filament organization | 1.0e-10 | 6.1e-07 |
| GO:1901653 | cellular response to peptide | 1.3e-10 | 7.4e-07 |
| GO:1901652 | response to peptide | 1.3e-10 | 7.6e-07 |
| GO:0001936 | regulation of endothelial cell proliferation | 3.1e-10 | 1.8e-06 |
| GO:0006913 | nucleocytoplasmic transport | 3.5e-10 | 2.0e-06 |
| GO:0051169 | nuclear transport | 3.5e-10 | 2.0e-06 |
| GO:0034248 | regulation of amide metabolic process | 4.4e-10 | 2.6e-06 |
| GO:0060485 | mesenchyme development | 7.0e-10 | 4.1e-06 |
| GO:0007044 | cell-substrate junction assembly | 7.5e-10 | 4.5e-06 |
| GO:0034504 | protein localization to nucleus | 1.5e-09 | 8.6e-06 |
| GO:0051493 | regulation of cytoskeleton organization | 3.5e-09 | 2.1e-05 |
| GO:0016032 | viral process | 5.4e-09 | 3.2e-05 |
| GO:0060840 | artery development | 6.7e-09 | 3.9e-05 |
| GO:0042060 | wound healing | 1.1e-08 | 6.7e-05 |
| GO:0032970 | regulation of actin filament-based process | 1.2e-08 | 7.2e-05 |
| O:0043086 | negative regulation of catalytic activity | 1.4e-08 | 8.4e-05 |
| GO:0034446 | substrate adhesion-dependent cell spreading | 1.4e-08 | 8.4e-05 |
| GO:0048193 | Golgi vesicle transport | 2.0e-08 | 1.2e-04 |
| GO:0048660 | regulation of smooth muscle cell proliferation | 2.0e-08 | 1.2e-04 |
| GO:0051345 | positive regulation of hydrolase activity | 2.3e-08 | 1.4e-04 |
| GO:0031331 | positive regulation of cellular catabolic process | 3.2e-08 | 1.9e-04 |
| GO:1903311 | regulation of mRNA metabolic process | 3.5e-08 | 2.1e-04 |
| GO:0001701 | in utero embryonic development | 3.9e-08 | 2.3e-04 |
| GO:0033002 | muscle cell proliferation | 4.2e-08 | 2.5e-04 |
| GO:0098727 | maintenance of cell number | 4.3e-08 | 2.6e-04 |
| GO:0016055 | Wnt signaling pathway | 4.7e-08 | 2.7e-04 |
| GO:0032835 | glomerulus development | 4.7e-08 | 2.8e-04 |
| GO:0150116 | regulation of cell-substrate junction organization | 4.7e-08 | 2.8e-04 |
| GO:0061448 | connective tissue development | 4.9e-08 | 2.9e-04 |
| GO:0019827 | stem cell population maintenance | 5.6e-08 | 3.3e-04 |
| GO:0198738 | cell-cell signaling by wnt | 5.8e-08 | 3.4e-04 |
| GO:0043161 | proteasome-mediated ubiquitin-dependent protein catabolic process | 7.0e-08 | 4.2e-04 |
| GO:0048872 | homeostasis of number of cells | 7.5e-08 | 4.5e-04 |
| GO:0006403 | RNA localization | 8.4e-08 | 4.9e-04 |
| GO:0001569 | branching involved in blood vessel morphogenesis | 9.6e-08 | 5.7e-04 |
| GO:0035924 | cellular response to vascular endothelial growth factor stimulus | 1.2e-07 | 6.9e-04 |
| GO:0034063 | stress granule assembly | 1.3e-07 | 7.6e-04 |
| GO:0006606 | protein import into nucleus | 1.3e-07 | 7.8e-04 |
| GO:0030099 | myeloid cell differentiation | 1.4e-07 | 8.2e-04 |
| GO:0050657 | nucleic acid transport | 1.7e-07 | 9.9e-04 |
| GO:0050658 | RNA transport | 1.7e-07 | 9.9e-04 |
| GO:0007162 | negative regulation of cell adhesion | 2.0e-07 | 1.2e-03 |
| GO:0044089 | positive regulation of cellular component biogenesis | 2.2e-07 | 1.3e-03 |
| GO:0070828 | heterochromatin organization | 2.3e-07 | 1.4e-03 |
| GO:0050767 | regulation of neurogenesis | 2.4e-07 | 1.4e-03 |
| GO:0045601 | regulation of endothelial cell differentiation | 2.7e-07 | 1.6e-03 |
| GO:0006397 | mRNA processing | 3.3e-07 | 1.9e-03 |
| GO:1900180 | regulation of protein localization to nucleus | 3.5e-07 | 2.1e-03 |
| GO:0071383 | cellular response to steroid hormone stimulus | 4.6e-07 | 2.7e-03 |
| GO:0061437 | renal system vasculature development | 5.5e-07 | 3.2e-03 |
| GO:0006402 | mRNA catabolic process | 5.6e-07 | 3.3e-03 |
| GO:1904019 | epithelial cell apoptotic process | 6.0e-07 | 3.6e-03 |
| GO:0071496 | cellular response to external stimulus | 6.6e-07 | 3.9e-03 |
| GO:0072594 | establishment of protein localization to organelle | 6.6e-07 | 3.9e-03 |
| GO:0051893 | regulation of focal adhesion assembly | 7.3e-07 | 4.3e-03 |
| GO:0090109 | regulation of cell-substrate junction assembly | 7.3e-07 | 4.3e-03 |
| GO:0048511 | rhythmic process | 8.5e-07 | 5.0e-03 |
| GO:0031507 | heterochromatin formation | 9.4e-07 | 5.6e-03 |
| GO:0010975 | regulation of neuron projection development | 1.0e-06 | 6.0e-03 |
| GO:0034976 | response to endoplasmic reticulum stress | 1.0e-06 | 6.1e-03 |
| GO:0009615 | response to virus | 1.1e-06 | 6.8e-03 |
| GO:0030032 | lamellipodium assembly | 1.2e-06 | 6.9e-03 |
| GO:0010586 | miRNA metabolic process | 1.3e-06 | 7.7e-03 |
| GO:0086103 | G protein-coupled receptor signaling pathway involved in heart process | 1.3e-06 | 7.8e-03 |
| GO:0003018 | vascular process in circulatory system | 1.3e-06 | 7.9e-03 |
| GO:0036294 | cellular response to decreased oxygen levels | 1.5e-06 | 8.6e-03 |
| GO:1904035 | regulation of epithelial cell apoptotic process | 1.6e-06 | 9.7e-03 |
| GO:0006470 | protein dephosphorylation | 1.7e-06 | 9.9e-03 |
| GO:0043065 | positive regulation of apoptotic process | 1.9e-06 | 1.1e-02 |
| GO:0018105 | peptidyl-serine phosphorylation | 2.0e-06 | 1.2e-02 |
| GO:0051145 | smooth muscle cell differentiation | 2.2e-06 | 1.3e-02 |
| GO:0031346 | positive regulation of cell projection organization | 2.4e-06 | 1.4e-02 |
| GO:0045814 | negative regulation of gene expression, epigenetic | 2.4e-06 | 1.4e-02 |
| GO:1902903 | regulation of supramolecular fiber organization | 3.0e-06 | 1.8e-02 |
| GO:0032386 | regulation of intracellular transport | 3.1e-06 | 1.8e-02 |
| GO:0050878 | regulation of body fluid levels | 3.1e-06 | 1.9e-02 |
| GO:0033044 | regulation of chromosome organization | 3.2e-06 | 1.9e-02 |
| GO:1990830 | cellular response to leukemia inhibitory factor | 3.3e-06 | 1.9e-02 |
| GO:0043087 | regulation of GTPase activity | 3.3e-06 | 1.9e-02 |
| GO:0000209 | protein polyubiquitination | 3.4e-06 | 2.0e-02 |
| GO:0051607 | defense response to virus | 3.9e-06 | 2.3e-02 |
| GO:0097581 | lamellipodium organization | 4.1e-06 | 2.4e-02 |
| GO:0000082 | G1/S transition of mitotic cell cycle | 5.1e-06 | 3.0e-02 |
| GO:1990823 | response to leukemia inhibitory factor | 5.2e-06 | 3.0e-02 |
| GO:0009791 | post-embryonic development | 5.2e-06 | 3.1e-02 |
| GO:0048010 | vascular endothelial growth factor receptor signaling pathway | 5.6e-06 | 3.3e-02 |
| GO:0038084 | vascular endothelial growth factor signaling pathway | 5.8e-06 | 3.4e-02 |
| GO:0045862 | positive regulation of proteolysis | 6.5e-06 | 3.8e-02 |
| GO:0006413 | translational initiation | 6.6e-06 | 3.9e-02 |
| GO:0016050 | vesicle organization | 7.4e-06 | 4.4e-02 |
| GO:0061614 | miRNA transcription | 7.6e-06 | 4.5e-02 |
| GO:0051347 | positive regulation of transferase activity | 8.0e-06 | 4.7e-02 |

**Results from BRCA data analysis**

**Table S14**. Top 25 differentially expressed genes from the grey multi-omic relation in the BRCA cohort, ranked by statistical significance. The table includes the feature name, test statistic estimate, p-value, and direction of change compared to unrelated participants.

| **feature** | **test_estimate** | **p_value** | **direction** | **p.adjust** |
| --- | --- | --- | --- | --- |
| SLC7A8 | 3.09 | 6.74e-25 | downregulated | 1.02e-22 |
| COL4A3BP | 1.34 | 1.28e-20 | downregulated | 1.95e-18 |
| KIAA1370 | 2.22 | 1.55e-19 | downregulated | 2.36e-17 |
| TBC1D9 | 3.78 | 1.27e-17 | downregulated | 1.93e-15 |
| ALAD | 1.36 | 1.31e-17 | downregulated | 1.99e-15 |
| SLC40A1 | 3.17 | 5.84e-17 | downregulated | 8.88e-15 |
| CKS1B | -1.39 | 4.24e-15 | upregulated | 6.44e-13 |
| CCNE1 | -3.34 | 6.02e-15 | upregulated | 9.14e-13 |
| UCK2 | -1.43 | 6.11e-15 | upregulated | 9.28e-13 |
| RAI2 | 3.07 | 1.18e-14 | downregulated | 1.79e-12 |
| NFE2L3 | -2.06 | 2.00e-14 | upregulated | 3.04e-12 |
| C6orf97 | 4.41 | 2.69e-14 | downregulated | 4.09e-12 |
| RHOB | 2.13 | 4.14e-14 | downregulated | 6.29e-12 |
| ABLIM3 | 3.41 | 6.14e-14 | downregulated | 9.33e-12 |
| SCUBE2 | 5.47 | 6.68e-14 | downregulated | 1.02e-11 |
| CA12 | 4.31 | 8.37e-14 | downregulated | 1.27e-11 |
| APH1B | 1.65 | 1.09e-13 | downregulated | 1.66e-11 |
| SLC7A2 | 4.41 | 1.25e-13 | downregulated | 1.90e-11 |
| TPCN1 | 1.38 | 1.38e-13 | downregulated | 2.10e-11 |
| KIAA0232 | 1.36 | 2.70e-13 | downregulated | 4.10e-11 |
| C10orf32 | 1.48 | 2.77e-13 | downregulated | 4.22e-11 |
| XBP1 | 2.62 | 2.94e-13 | downregulated | 4.47e-11 |
| CBX2 | -2.71 | 3.04e-13 | upregulated | 4.62e-11 |
| FAM123B | -1.49 | 3.42e-13 | upregulated | 5.20e-11 |
| CBS | -3.24 | 3.47e-13 | upregulated | 5.28e-11 |

**Table S15**. List of differentially expressed proteins from the grey multi-omic relation in the BRCA cohort, ranked by statistical significance. The table includes the feature name, test statistic estimate, p-value, and direction of change compared to unrelated participants.

| **feature** | **test_estimate** | **p_value** | **direction** | **p.adjust** |
| --- | --- | --- | --- | --- |
| Cyclin_B1 | -1.46 | 1.31e-19 | upregulated | 1.44e-18 |
| MSH6 | -1.12 | 2.06e-14 | upregulated | 2.26e-13 |
| ASNS | -0.80 | 2.13e-13 | upregulated | 2.35e-12 |
| Chk2 | -0.66 | 2.20e-11 | upregulated | 2.42e-10 |
| MSH2 | -0.85 | 1.14e-10 | upregulated | 1.26e-09 |
| Cyclin_E1 | -0.77 | 1.04e-08 | upregulated | 1.15e-07 |
| FoxM1 | -0.94 | 1.13e-08 | upregulated | 1.25e-07 |
| GAPDH | -1.18 | 2.90e-06 | upregulated | 3.19e-05 |
| PREX1 | 0.50 | 1.97e-04 | downregulated | 2.17e-03 |
| TFRC | -0.60 | 7.54e-04 | upregulated | 8.30e-03 |
| S6_pS240_S244 | -0.48 | 3.10e-03 | upregulated | 3.42e-02 |


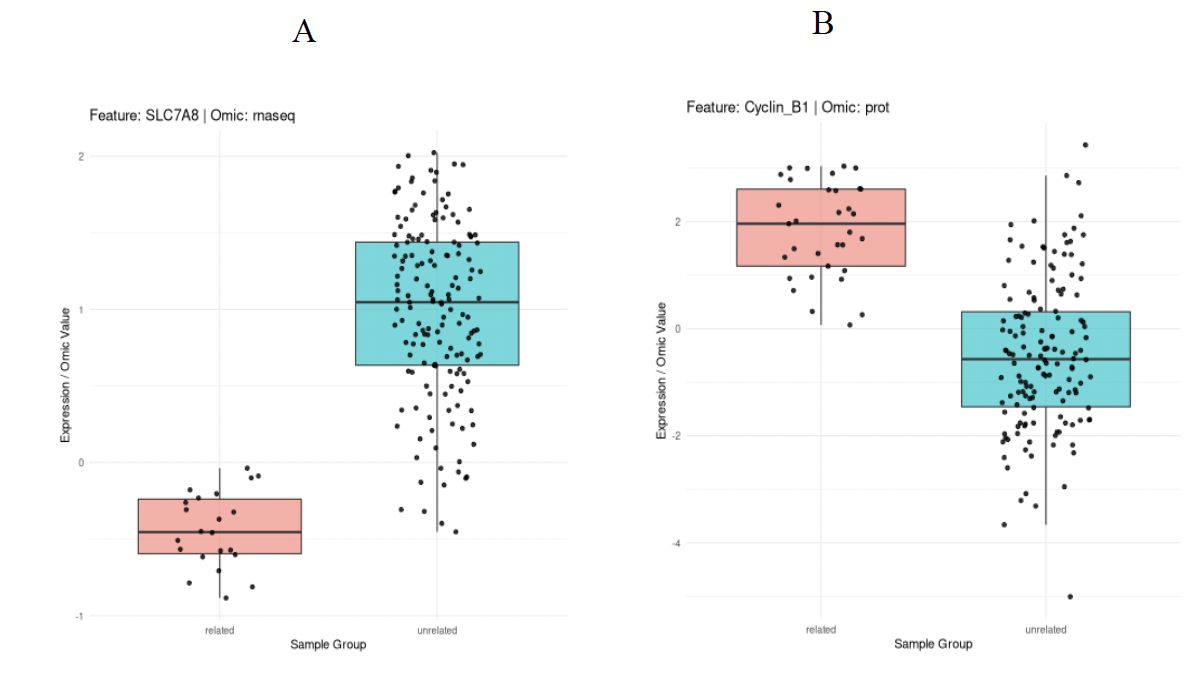


**Figure S4**. Boxplots showing differences in top-ranked: **A)** mRNA expression, and **B)** protein expression levels of the participants within the grey multi-omic relation in the BRCA cohort between (‘related’) and those outside the relation in the rest of the cohort (‘unrelated’).

1. **Benchmarking MOBAA against state-of-the-art methods on multi-omics data from KIPAN**

We evaluated each method’s ability to recover the known subtypes by examining cluster/factor composition and performing one-sided proportion tests for subtype enrichment. A low p-value indicates that a cluster or factor contains significantly more samples from a subtype than expected by chance. Our comparative evaluation demonstrates that all three integrative methods were effective in identifying the known KICH, KIRC, and KIRP subtypes within the TCGA KIPAN dataset, yet each achieved this through distinct operational strengths. MOBAA generated three highly pure subtype-aligned clusters with no cross-contamination, offering exceptional specificity but limited subtype coverage, thereby highlighting its suitability for high-confidence subtype core detection. Conversely, iClusterPlus clustered nearly all samples, not only distinguishing primary subtype boundaries but also uncovering multiple clusters within each subtype, indicative of deeper biological heterogeneity; however, this broader sensitivity occasionally produced mixed clusters, reflecting potential oversplitting. MOFA2, while not intended as a direct clustering tool, captured the major axes of variation driving subtype differences through latent factors, enabling subtype differentiation through factor combinations rather than single discrete clusters. As such, MOFA2 provides superior interpretability of underlying variance patterns at the cost of less immediate cluster purity. Overall, these findings emphasize that method selection should depend on whether the objective prioritizes cluster purity (MOBAA), full-sample classification and heterogeneity detection (iClusterPlus), or mechanistic interpretability through continuous latent structure (MOFA2).

In pathway analysis, MOFA2 and MOBAA identified a number of significant pathways per subtype and captured known hallmarks more comprehensively than iClusterPlus (Pandey et al., 2020). For example, in KICH, MOFA2 and MOBAA both highlighted widespread metabolic alterations (enriched pathways including OXPHOS and amino acid metabolism), whereas iClusterPlus yielded only 10 significant terms, missing the OXPHOS signature (it instead emphasized immune/ciliary processes). In KIRC, MOFA2/MOBAA detected numerous pathways (e.g. hypoxia–glycolysis and immune/inflammatory signals) reflecting VHL/HIF-driven biology, while iClusterPlus found only 1–2 pathways at FDR significance (primarily a developmental signal). For KIRP, all methods captured the extensive metabolic reprogramming; iClusterPlus actually reported an especially large set of ~50–60 catabolic pathways (due to the strong metabolic gene signal), and MOFA2/MOBAA similarly returned a broad metabolic profile for KIRP (including mitochondrial translation, TCA cycle, etc., many in common with iCluster). MOBAA (which integrates methylation and expression) showed performance comparable to MOFA2 – it found many of the same relevant pathways and added some unique ones. Notably, MOBAA flagged immune-response pathways in KICH via methylation data (e.g. LPS/bacterial response), hinting at tumor–microenvironment interactions that the other methods did not highlight. This suggests MOBAA’s integrative design can broaden findings (capturing both genetic and epigenetic dysregulation). In terms of quantity, per subtype MOFA2 and MOBAA each returned dozens of significant pathways, whereas iClusterPlus tended to return fewer (especially for clear cell) or somewhat different ones. In terms of biological relevance, the pathways from MOFA2 and MOBAA were highly concordant with known subtype biology (e.g. MOFA2/MOBAA correctly emphasized chromophobe’s oxidative metabolism and clear cell’s HIF-related processes), while iClusterPlus sometimes missed these emphases. The subtype recovery performance of each method including the identified clusters/factors and their composition is presented in Table S14.

**Table S16**. Summary of subtype-enriched clusters (or factors) identified by each method on KIPAN dataset. Each cell shows the count of clusters significantly enriched for a given subtype, with an indication of purity and p-values. Sample counts per factor/module reflect the subset of high-loading samples used to define the factor/module, not the full subtype population.

| **Method** | **KICH** **(63/566 samples)**  **(Specificity, p-value)** | **KIRC (291/566 samples)**  **(Specificity, p-value)** | **KIRP (212/566 samples)**  **(Specificity, p-value)** |
| --- | --- | --- | --- |
| **MOBAA** | Recovered 1 module with 100% samples (30/30) from KICH subtype (pure KICH, *p*-value < 2.2e-16). | Recovered 1 module with 100% samples (17/17) from KIRC subtype (pure KIRC, *p*-value = 8.3e-5). | Recovered 1 module with 100% samples (45/45) from KIRP subtype (pure KIRP, *p*-value < 2.2e-16). |
| **iClusterPlus** | Recovered 3 clusters (1 pure, 2 mixed). One cluster had 33/34 KICH (*p*-value < 2.2e-16); two clusters had ~33–39% KICH (*p*-values ~3.6e-5 to 2.4e-7). | Recovered 3 clusters (2 pure, 1 mixed). Two large clusters had ~95–99% KIRC (*p*-value = 3.3e-16 and 9e-16); one cluster with ~89% KIRC (*p*-value = 2.9e-6) | Recovered 4 clusters (3 pure, 1 mixed). Three clusters had ~85–100% KIRP (*p*-value = 7e-6, 1.4e-15, <2.2e-16); one cluster ~52% KIRP (*p*-value=0.04) |
| **MOFA2** | Recovered 4 factors (all mixed). Each factor captured 20–60% KICH. Two factors were strongly enriched (p-value < 2.2e-16); two had moderate enrichment (*p*-value=0.01, 0.005). | Recovered 2 factors (mixed) with ~60–70% KIRC in each. Both showed significant enrichment (*p*-value=6e-4 and 7e-3). Other factors also had KIRC members but not above random expectation. | Recovered 4 factors (1 near-pure, 3 mixed). One factor was 86% KIRP (*p*-value = 5.4e-14); three factors with ~50–55% KIRP (*p*-values=6e-3, 1e-2, 0.03) |

1. **Benchmarking MOBAA against state-of-the-art methods on multi-omics data from BRCA**

We also evaluated each method’s ability to recover the known BRCA subtypes by examining cluster/factor composition and performing one-sided proportion tests for subtype enrichment. Here again, all three integrative methods were effective in identifying the known Basal-like, HER2-enriched, Luminal A, Luminal B, and Normal-like subtypes within the TCGA BRCA dataset through distinct operational strengths. MOBAA generated a single highly pure Basal-like cluster with no cross-contamination (16/16 Basal-like samples, p-value = 2.6e-11), offering exceptional specificity but limited subtype coverage. No distinct clusters were recovered for the other subtypes, highlighting MOBAA’s strength in high-confidence core subtype detection but also its limitation in comprehensively capturing the full spectrum of subtype diversity in this dataset. Conversely, iClusterPlus clustered nearly all samples, not only distinguishing primary subtype boundaries but also uncovering multiple clusters within each subtype, indicative of deeper biological heterogeneity. For example, it recovered two Basal-like clusters, one pure (12/12 Basal-like, p-value = 1.1e-08) and one mixed (16/17 Basal-like, ~94%, p-value = 3.5e-10), as well as multiple mixed clusters for Luminal A and Luminal B, each with varying degrees of subtype enrichment. However, this broader sensitivity occasionally produced mixed clusters, particularly for HER2-enriched and Normal-like subtypes, reflecting potential oversplitting and reduced cluster purity.

MOFA2 recovered a pure Basal-like factor (100% Basal-like, p-value = 1.2e-12) and a second factor with ~67% Basal-like enrichment (p-value = 5.6e-05), demonstrating strong alignment with this subtype. For Luminal B, MOFA2 identified two moderately enriched factors (50% and ~44% Luminal B, p-values = 0.006 and 0.02, respectively), while HER2-enriched and Normal-like subtypes were not distinctly captured. As such, MOFA2 provides superior interpretability of underlying variance patterns at the cost of less immediate cluster purity and limited resolution for smaller or less distinct subtypes. Although MOBAA identified statistically significant genes in the BRCA dataset, these genes did not map to any enriched biological pathways. As a result, we did not perform pathway analysis with the other methods, since meaningful comparison at the pathway level would not have been possible in the absence of a common interpretive baseline. Overall, these findings emphasize that method selection should depend on whether the objective prioritizes cluster purity (MOBAA), full-sample classification and heterogeneity detection (iClusterPlus), or mechanistic interpretability through continuous latent structure (MOFA2).

**Table S17**. Summary of subtype-enriched clusters (or factors) identified by each method on BRCA dataset. Each cell shows the count of clusters significantly enriched for a given subtype, with an indication of purity and p-values. Sample counts per factor/module reflect the subset of high-loading samples used to define the factor/module, not the full subtype population.

| **Method** | **Basal-like (39/179 samples)**  **(Specificity, p-value)** | **HER2-enriched (12/179 samples)**  **(Specificity, p-value)** | **Luminal A (85/179 samples)**  **(Specificity, p-value)** | **Luminal B (38/179 samples)**  **(Specificity, p-value)** | **Normal-like (5/179 samples)**  **(Specificity, p-value)** |
| --- | --- | --- | --- | --- | --- |
| **MOBAA** | Recovered 1 pure module with 100% Basal-like samples (16/16) (*p*-value = 2.6e-11). | None identified. | None identified. | None identified. | None identified. |
| **iClusterPlus** | Recovered 1 pure and 1 mixed cluster.  The pure cluster had 12/12 Basal-like samples (*p*-values ~1.1e-08).  The mixed cluster had 16/17 (~94%) Basal-like samples (*p*-value = 3.5e-10). | Recovered 1 mixed cluster with 4/13 (~31%) HER2 samples (*p*-value = 0.009). | Recovered 2 mixed clusters. One had 10/11 (~91%) (*p*-value = 0.004) and the other had 17/25 (~68%) (*p*-value = 0.03) Luminal A samples. | Recovered 2 mixed clusters. One had 9/16 (~56%) (*p*-value = 0.002) and the other had 10/14 (~71%) (*p*-value = 7.9e-05) Luminal B samples. | Recovered 1 mixed cluster with 2/9 (~22%) Normal-like samples (*p*-value = 0.02). |
| **MOFA2** | Recovered 2 factors (1 pure, 1 mixed). The pure factor captured 18/18 (100%) Basal-like samples (p-value = 1.2e-12). The mixed factor captured 12/18 (~67%) of the Basal-like samples (p-value = 5.6e-05). | Recovered one mixed factor with 5/18 (~28%) HER2-enruched samples (*p*-value=0.006). | None identified. | Recovered 2 mixed factors: one with 9/18 (50%) Luminal B samples (p-value=0.006), and the other with 8/18 (~44%) Luminal B samples (p-value = 0.02) | None identified. |

1. **Benchmarking MOBAA against state-of-the-art methods on simulated overlapping multi-omics data**

Here we evaluated each method’s ability to recover the known subtypes on simulated overlapping multi-omics data. The subtypes were A, B, and C containing 69, 57, and 74 samples, respectively.

MOBAA identified several multi-omic modules that aligned strongly with the known subtypes. For subtype A, a “brown” module contained 19 samples, all belonging to subtype A (100% purity; *p*-value = 4.1×10⁻⁹), capturing about 28% of all A samples and indicating that MOBAA recovered a core subset while the remaining A samples were distributed across other modules. Subtype B was almost perfectly recovered in a single “turquoise” module containing 56 samples, all subtype B (~100% purity; p < 2.2×10⁻¹⁶), representing 98% of all B samples. Subtype C corresponded to a “blue” module with 29 samples, all subtype C (*p*-value = 4.1×10⁻¹²), covering roughly 39% of the subtype. In addition, MOBAA produced a pure “grey” module of 17 samples associated with the remaining subtype (*p*-value = 8.3×10⁻⁵). Overall, MOBAA recovered a perfectly pure module for each subtype, although A and C were only partially captured in single modules.

MOFA2 produced latent factors representing major axes of multi-omic variation. Factor 2 showed a clear subtype B–specific signal, with 20 high-loading samples all belonging to subtype B (*p*-value = 4.1×10⁻¹²), accounting for roughly 35% of the subtype. Factor 1 captured 20 samples from subtype C with 100% purity (*p*-value = 1.0×10⁻⁸), covering about 27% of C. In contrast, no latent factor was significantly enriched for subtype A, indicating that MOFA2 did not isolate this subtype within a single latent dimension. Overall, MOFA2 identified strong subtype-associated factors for B and C, but not for A.

iClusterPlus produced multiple highly pure clusters for each subtype, reflecting a finer-grained structure in the data. For subtype A, six clusters containing 9–11 samples each were composed entirely of A samples (*p*-value = 10⁻⁵), together accounting for nearly all 69 A samples. Subtype B was represented by four pure clusters of 6–18 samples (*p*-value = 10⁻⁹–10⁻¹¹), collectively covering all 57 B samples. Similarly, subtype C appeared in four pure clusters of 16–23 samples (*p*-value = 10⁻⁷–10⁻¹⁰), capturing all 74 C samples. Thus, while iClusterPlus achieved perfect subtype purity, it fragmented each subtype into multiple smaller clusters, likely reflecting either genuine within-subtype heterogeneity or over-splitting driven by specifying more clusters than the true number of subtypes.

Overall, all three methods strongly recovered subtypes B and C, with highly significant enrichment (*p*-value < 0.001). For subtype A, MOBAA and iClusterPlus both produced A-enriched clusters (*p*-value < 10⁻⁸ and ~10⁻⁵, respectively), whereas MOFA2 did not identify an A-specific factor. MOFA2 captured the core signals of B and C with single latent factors, while MOBAA partially captured A and C in distinct modules, and iClusterPlus recovered all subtypes but split each into multiple pure subclusters. The subtype recovery performance of each method including the identified clusters/factors and their composition is presented in Table S15.

**Table S18**. Summary of subtype-enriched clusters (or factors) identified by each method on simulated overlapping multi-omics data. Each cell shows the count of clusters significantly enriched for a given subtype, with an indication of purity and p-values.

| **Method** | **Subtype A (69/200 samples)**  **(Specificity, p-value)** | **Subtype B (57/200 samples)**  **(Specificity, p-value)** | **Subtype C (74/200 samples)**  **(Specificity, p-value)** |
| --- | --- | --- | --- |
| **MOBAA** | Recovered 1 module with 100% samples (19/19) from subtype A. Enrichment *p*-value = 4.1e-09. | Recovered 1 module with 100% samples (56/56) from subtype B. Enrichment *p*-value < 2.2e-16. | Recovered 1 module with  100% samples (29/29) from subtype C. Enrichment *p*-value = 4.1e-12. |
| **iClusterPlus** | Recovered 6 clusters (sizes 9–11) all containing 100% samples from subtype A.  Each *p*-value~10^−5^. | Recovered 4 clusters (sizes 6–18) all containing 100% samples from subtype B.  *P*-value ~10^−9^ to 10^−11^ each. | Recovered 4 clusters (sizes 16–23) all containing 100% samples from subtype C.  *P*-value ~10^−7^ to 10^−10^ each. |
| **MOFA2** | Not distinct: No factor exclusively for subtype A.  (Subtype A samples distributed across factors; no *p*-value < 0.05) | Recovered 1 factor (Factor 2) with 20/20 (100%) samples from subtype B.  Enrichment *p*-value = 4.1e-12. | Recovered 1 factor (Factor 1) with 20/20 (100%) samples from subtype C.  Enrichment *p*-value = 1.0e-08. |
